# Native-Invasive Plants vs. Halophytes in Mediterranean Salt Marshes: Stress Tolerance Mechanisms in Two Related Species

**DOI:** 10.3389/fpls.2016.00473

**Published:** 2016-04-18

**Authors:** Mohamad Al Hassan, Juliana Chaura, María P. López-Gresa, Orsolya Borsai, Enrico Daniso, María P. Donat-Torres, Olga Mayoral, Oscar Vicente, Monica Boscaiu

**Affiliations:** ^1^Instituto de Biología Molecular y Celular de Plantas, (UPV-CSIC), Universitat Politècnica de ValènciaValencia, Spain; ^2^Instituto de Investigación para la Gestión Integrada de Zonas Costeras, Universitat Politècnica de València, Grau de GandíaValencia, Spain; ^3^Departamento de Didáctica de las Ciencia Experimentales y Sociales, Universitat de ValènciaValencia, Spain; ^4^Instituto Agroforestal Mediterráneo, Universitat Politècnica de ValènciaValencia, Spain

**Keywords:** *Dittrichia viscosa*, *Inula crithmoides*, native-invasive species, salinity tolerance, water stress tolerance, osmolytes, toxic ions, oxidative stress

## Abstract

*Dittrichia viscosa* is a Mediterranean ruderal species that over the last decades has expanded into new habitats, including coastal salt marshes, ecosystems that are *per se* fragile and threatened by human activities. To assess the potential risk that this native-invasive species represents for the genuine salt marsh vegetation, we compared its distribution with that of *Inula crithmoides*, a taxonomically related halophyte, in three salt marshes located in “La Albufera” Natural Park, near the city of Valencia (East Spain). The presence of *D. viscosa* was restricted to areas of low and moderate salinity, while *I. crithmoides* was also present in the most saline zones of the salt marshes. Analyses of the responses of the two species to salt and water stress treatments in controlled experiments revealed that both activate the same physiological stress tolerance mechanisms, based essentially on the transport of toxic ions to the leaves—where they are presumably compartmentalized in vacuoles—and the accumulation of specific osmolytes for osmotic adjustment. The two species differ in the efficiency of those mechanisms: salt-induced increases in Na^+^ and Cl^−^ contents were higher in *I. crithmoides* than in *D. viscosa*, and the osmolytes (especially glycine betaine, but also arabinose, fructose and glucose) accumulated at higher levels in the former species. This explains the (slightly) higher stress tolerance of *I. crithmoides*, as compared to *D. viscosa*, established from growth inhibition measurements and their distribution in nature. The possible activation of K^+^ transport to the leaves under high salinity conditions may also contribute to salt tolerance in *I. crithmoides*. Oxidative stress level—estimated from malondialdehyde accumulation—was higher in the less tolerant *D. viscosa*, which consequently activated antioxidant responses as a defense mechanism against stress; these responses were weaker or absent in the more tolerant *I. crithmoides*. Based on these results, we concluded that although *D. viscosa* cannot directly compete with true halophytes in highly saline environments, it is nevertheless quite stress tolerant and therefore represents a threat for the vegetation located on the salt marshes borders, where several endemic and threatened species are found in the area of study.

## Introduction

Salt marshes are coastal ecosystems developed in temperate zones, occupied mainly by halophytic vegetation that can be exposed, in some cases, to tidal flooding. These are specialized habitats, characterized by a high primary productivity and species diversity, which support a wide variety of native flora and fauna, and constitute as well important areas for wintering aquatic birds (Simas et al., 2001). These ecosystems are economically important since they can be used as nursery grounds for several fish and crustacean fisheries (Dijkema et al., 1990; Reed, 1990). Salt marshes are among the most abundant, fertile, and accessible habitats on earth, and therefore are highly threatened by human activities (industrial pollution, urbanization, agriculture, etc.), which have damaged many existing salt marshes in the world (Bromberg Gedan et al., 2009). In the SE Iberian Peninsula, the situation of these habitats is particularly critical. Many of them were destroyed in the past, due to their transformation into cropland or by desiccation for fear of malaria. In the Valencia region (Spain), where this study has been carried out, the coastline supports virtually all farming, much of industrial activity, and shelters large population centers. This, along with a huge pressure from tourism, produced a high impact on natural ecosystems, including salt marshes. The situation has changed in recent years, when the ecological value of such habitats started to be considered. All coastal lagoons in the area have been proposed as SCIs (Sites of Community Interest) to join the Natura 2000 network as SACs (Special Areas of Conservation) and SPAs (Special Protection Areas for birds) and catalogued as priority habitats in the region of Valencia (Laguna, 2003). In particular, the habitats 1150 (Coastal lagoons) and 1510 (Mediterranean salt steppes, *Limonietalia*), intrinsically related to the salt marshes, are considered priority habitats by the European legislation (Council Directive, 1992). In addition, in such areas many Plant Micro-Reserves have been declared, some devoted to the protection of halophytic vegetation (Fos et al., 2014). These ecosystems house a characteristic flora of halophytes, which have developed different strategies to adapt to salt stress and include some taxa of special interest, being endemic or threatened; the species distributions are shaped by physical and chemical gradients in the environment, but also by biological interactions (Adams, 1963; Lefor et al., 1987). The high specialization of the organisms living in salt marshes contributes to the vulnerability of these habitats. Among numerous threats, the pressure of invasive plants has strong effects in such fragmented and linear ecosystems (Petillón et al., 2005, and references therein).

The term “invasive” is usually employed for alien (synonymous to exotic, non-indigenous, introduced, or newcomer) species which have the ability to spread aggressively outside their natural range and are potentially dangerous for the environment, economy, or human health (Callaway and Aschehoug, 2000; Byers et al., 2002; Hejda et al., 2009). The term invasive is also accepted in a broader sense, including indigenous or native species that occupy new ecosystems, which they alter (Mack, 1985; Gouyon, 1990; Le Floch et al., 1990; Carey et al., 2012; Muñoz-Vallés and Cambrollé, 2015). When invasive species show increased abundance, density or geographic extent, this may be considered as potentially problematic (Richardson and Pyšek, 2004). The mechanism of invasion is based on the opening of novel niches or through the extension of pre-existing ones (Shea and Chesson, 2002; Valéry et al., 2008), and invasive species may eliminate autochthonous plants from their natural habitats, due to their high competitiveness. Weedy native species sometimes have major impacts and are the subject of intensive and expensive management efforts (Williamson, 1998). In this regard, the role of native species as a potential threat for biodiversity conservation is an emerging problem, which is still under discussion. These autochthonous species, which induce notable changes in the environment by undergoing rapid expansion, are known as “native-invasive” species. In SW Spain, for example, native *Retama monosperma* has become invasive in sand dunes, causing significant damage to the natural ecosystem, even greater than that due to alien invasive species under similar climatic conditions (Muñoz Vallés et al., 2011).

The genus *Dittrichia* Greuter has a wide Mediterranean distribution and shows very close relation to the genus *Inula* L., differing in morphologic characteristics of the achenes and pappus-haire (Brullo and de Marco, 2000). *Dittrichia viscosa* (L.) Greuter, formerly ascribed to the genus *Inula* as *I. viscosa* (L.) Aiton, is a perennial, 40-130 cm high plant, very common in the western Mediterranean but with penetration also in its eastern part. Its primary habitats are gravel riverbeds, mountain screes, and sandy and rocky coasts, but it is frequent mostly in secondary habitats as roadsides and abandoned fields, and sometimes in croplands as a weed (Brullo and de Marco, 2000). The species shows a remarkable pioneer character, and in the last decades largely expanded its range in the circum-Mediterranean countries, possibly due to increased human disturbances (Wacquant, 1990; Mateo et al., 2013). *D. viscosa* has very high coverage percentages in riparian plant communities in SE Spain, that have undergone major alterations and have been seriously degraded as a result of anthropic effects (Salinas et al., 2000); in a recent study, it has been also reported as the most frequent species on roadsides in Portugal (Simões et al., 2013). Its capability to colonize new habitats and threaten the biodiversity has been well documented (Wacquant, 1990) and was related to characteristics such as its phenotypic plasticity (Wacquant and Bouab, 1983), high stress tolerance (Curadi et al., 2005), and resistance to chemical pollution (Murciego et al., 2007; Fernández et al., 2013), as well as to its allelopathic effects (Omezzine et al., 2011). In the last 50 years, *D. viscosa* has become an invader in the NW Mediterranean region, since it increased its ecological range under disturbance pressure and is colonizing new habitats (Wacquant, 1990; Boonne et al., 1992; Wacquant and Baus Picard, 1992; Mateo et al., 2013). The species recent expansion in the Iberian Peninsula has also been correlated to temperature increases due to the accelerated global warming (Sobrino Vesperinas et al., 2001). *D. viscosa* has been catalogued as an invasive alien plant in the region of Asturias (N Spain), where it invades especially sensitive environments of high ecologic value (Castaño, 2007). It was occasionally reported in salt marshes in the Mediterranean region (Molinier and Tallon, 1970; Llorens, 1986; Korakis and Gerasimidis, 2006), and it became very frequent over the last decades in the area under study; nowadays it is present in most of the 10 × 10 km UTM squares (Mateo et al., 2013). It has been proposed that *D. viscosa* could be used as a secondary plant in biological pest control, which is a method to control pests without the application of potentially dangerous chemical pesticides (Parolin et al., 2014). This and other potential biotechnological applications of this species, such as its promising use for phytoremediation in mining-affected semiarid soils, since it is an efficient bioaccumulator of trace metals (Barbafieri et al., 2011; Jimenez et al., 2011; Pérez et al., 2012), could extend its distribution as a consequence of human uses.

The main aim of this work was to evaluate the potential risk that *D. viscosa* represents for Mediterranean salt marsh vegetation. To assess the degree of threat that *D. viscosa* may pose to the genuine halophytic vegetation in SE Spain, we have first analyzed its distribution in three salt marshes located in “La Albufera” Natural Park, Valencia, in relation to soil electric conductivity and moisture and in comparison with a closely related, typical halophyte, *Inula crithmoides* [syn. *Limbarda crithmoide*s (L.) Dumort]. This latter species is a small shrub 30-100 cm high, frequent on cliffs and salt marshes, with a Mediterranean-Atlantic distribution, reaching its northern limit in Scotland. The field work was complemented studying the responses of the two species to salt and water stress under controlled experimental conditions in a greenhouse, to establish the relative resistance to stress of the two species and the underlying tolerance mechanisms. For this, some specific biochemical stress markers associated with basic, conserved response pathways were determined in young plants of *D. viscosa* and *I. crithmoides* subjected to salt and water stress treatments. We quantified, specifically: (1) growth parameters, that would clearly indicate their relative tolerance to stress, (2) photosynthetic pigments (chlorophylls a and b and total carotenoids), (3) the major putative osmolytes (proline, glycine betaine, and soluble carbohydrates), (4) monovalent ions (Na^+^, K^+^, and Cl^−^), (5) levels of malondialdehyde (MDA), as an indicator of oxidative stress, (6) total antioxidant activity, by the DPPH radical scavenging assay, and total phenolics and flavonoids as examples of non-enzymatic antioxidants, and (7) antioxidant enzyme activities (superoxide dismutase, catalase, and glutathione reductase).

## Materials and methods

### Field study

**Figure 1:**
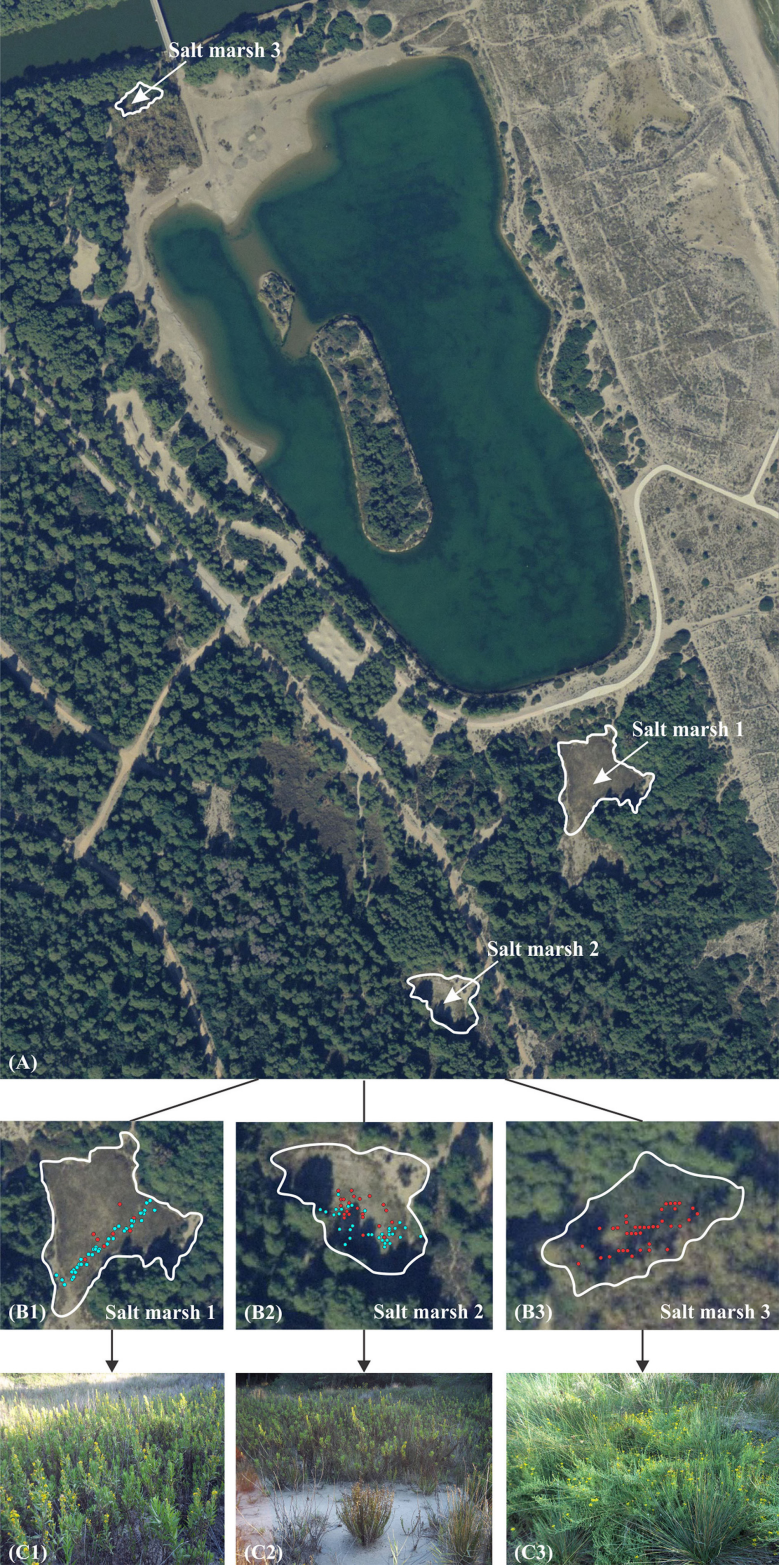
**General view of the area in “La Albufera” Natural Park where the field work was carried out, with the location of the three selected salt marshes (A)**. Distribution of the two species (*Dittrichia viscosa* in light blue and *Inula crithmoides* in red) in salt marsh 1, 2 and 3 (**B1–B3**, respectively). General aspect of the three salt marshes in autumn **(C1–C3)**.

The presence of *D. viscosa* and *I. crithmoides* plants in relation to soil electric conductivity (hereafter EC) and soil moisture was registered in three salt marshes located in “La Albufera” Natural Park (39°47′28″N, 1°04′25″W) near Valencia, in eastern Spain. La Albufera is the biggest lake in the Iberian Peninsula, formed by the gradual closure of an ancient marine gulf. The salt marshes appear in small inter-dune depressions located in the narrow land strip between the sea and the lake (Figure 1). Mediterranean salt marsh depressions usually appear to be endorheic. Sudden flooding caused by heavy rains coming in from the sea is usually followed by long droughts and periods of high concentration of salts (Quintana et al., 1998). The climate is typical Mediterranean, with a summer peak of temperature and absence of rainfall, resulting in periods of severe summer stress (Rivas-Martínez and Rivas-Sáenz, 1996-2009).

The presence of the two species was evaluated along a linear transect in the first salt marsh, since it is much extended and there is a high density of *D. viscosa* specimens. In the other two salt marshes, considerably smaller, soil EC was measured in each point where one of the two species appeared. Soil EC and moisture (%) were analyzed with a WET-2 Sensor (Delta–T Devices, UK) which allows direct *in situ* non-destructive measurements in the root zones of the plants. Coordinates of these points were established with a GPS (Garmin GPSMAP 76CSx). All field data were taken in spring 2015.

### Plant material

Mature floral heads of *D. viscosa* and *I. crithmoides* were sampled in one of the salt marshes, in October 2014, and well-shaped achenes were directly sown on a mixture of commercial peat and vermiculite (3:1). After 20 days, seedlings homogeneous in size were selected and placed in individual pots. Three weeks later, when young plants were robust enough, treatments were initiated. For the salt treatments, plants were watered twice a week with Hoagland nutritive solution (Hoagland and Arnon, 1950) containing NaCl at 75, 150, 300, or 600 mM final concentrations, or without salt for the non-stressed controls (1.5 L per tray, each containing 12 pots). For the water stress treatments, watering was completely stopped. All experiments were conducted in a controlled environment chamber, under the following conditions: long-day photoperiod (16 h of light), temperature of 23°C during the day and 17°C at night, ca. 300 ppm CO_2_ level, and 50-80% relative humidity. All measurements and biochemical assays were performed using leaf material, half of which was harvested after 3 weeks, and the other half after 6 weeks of salt treatment; in the case of water stress, only the 3-week samples were used, since not all plants survived the longer treatment. To better compare the effect of the stress treatments on plant growth for the two species, the fresh weight (hereafter FW) of the leaves of each sample was expressed as percentage of the FW of the corresponding non-treated control; the absolute FW values of the controls are indicated in the legend to **Figure 3**. Part of the leaf material was dried at 65°C until constant weight, to calculate the water content of the plants (WC), in percentage, as WC (%) = [(FW – DW)/ FW] × 100.

### Electrical conductivity of the soil in the pots

Electrical conductivity of the substrate was measured after 3 and 6 week-treatments. Soil samples from five pots per treatment were air-dried and then passed through a 2-mm sieve. For each sample, a soil:water (1:5) suspension was prepared in deionized water and mixed for 1 h at 600 rpm, and 21°C. Electric conductivity (EC_1:5_) was measured with a Crison Conductivity-meter 522 and expressed in dS m^−1^.

### Photosynthetic pigments

Total carotenoids, chlorophyll a (Chl a) and chlorophyll (Chl b) were measured following Lichtenthaler and Wellburn (1983): 100 mg of fresh leaf material was ground in the presence of 20 ml of ice-cold 80% acetone, mixed by vortexing and centrifuged. The supernatant was collected and its absorbance was measured at 663, 646, and 470 nm. The final values were expressed in mg g^−1^ DW.

### Osmolyte quantification

Glycine betaine (GB) was determined in dried leaf tissue according to Grieve and Grattan (1983). The sample (100 mg) was ground with 2 ml of Mili-Q water, and then extracted with 1, 2-dichlorethane; the absorbance of the solution was measured at a wavelength of 365 nm. GB concentration was expressed as μmol g^−1^ DW.

Proline (Pro) content was quantified using fresh leaf material, according to the ninhydrin-acetic acid method of Bates et al. (1973). Pro was extracted in 3% aqueous sulfosalicylic acid, the extract was mixed with acid ninhydrin solution, incubated for 1 h at 95°C, cooled on ice and then extracted with two volumes of toluene. Absorbance of the supernatant was read at 520 nm, using toluene as a blank. Pro concentration was expressed as μmol g^−1^ DW.

### Identification and quantification of soluble carbohydrates by HPLC

The water-soluble sugar fraction (mono and oligosaccharides) was analyzed using a Waters 1525 High Performance Liquid Chromatography system, coupled to a 2424 evaporative light scattering detector (ELSD). The source parameters of ELSD were the following: gain 75, data rate 1 point per second, nebulizer heating 60%, drift tube 50°C, and gas pressure 2.8 Kg/cm^2^. Analysis was carried out injecting 20 μL aliquots with a Waters 717 auto-sampler into a Prontosil 120-3-amino column (4.6 × 125 mm; 3 μm particle size) maintained at room temperature. An isocratic flux (1 mL/min) of 85% acetronitrile (J.T. Baker) during 25 min was applied in each run. Standards of glucose, fructose, and arabinose served to identify peaks by co-injection. Sugars were quantified by peak integration using the Waters Empower software and comparison with glucose, fructose, and arabinose standard calibration curves.

### Monovalent ions levels

Extractions were performed according to Weimberg (1987), by incubating the samples (0.15 g of dried and ground leaf material in 25 ml of water) for 1 h at 95°C in a water bath, followed by filtration through a filter paper (particle retention 8–12 μm). Sodium and potassium were quantified with a PFP7 flame photometer (Jenway Inc., Burlington, USA) and chlorides were measured using a Merck Spectroquant Nova 60® spectrophotometer and its associated test kit (Merck, Darmstadt, Germany).

### MDA and non-enzymatic antioxidants

Dried leaf material was extracted in 80% methanol, in a rocker shaker, for 24–48 h. Malondialdehyde (MDA) content in the extracts was determined according to the method described by Hodges et al. (1999). The samples were mixed with 0.5% thiobarbituric acid (TBA) prepared in 20% TCA (or with 20% TCA without TBA for the controls), and then incubated at 95°C for 20 min. After stopping the reaction, the absorbance of the supernatants was measured at 532 nm. The non-specific absorbance at 600 and 440 nm was subtracted and MDA concentration was determined using the equations described by Hodges et al. (1999).

Total antioxidant activity in the extracts, measured by their ability to quench the radical 2,2-diphenyl-1-picrylhydrazyl (DPPH), was determined spectrophotometrically according to Falchi et al. (2006). The methanol-soluble fraction was appropriately diluted with 96% ethanol, in a final volume of 2 mL, to which 0.5 mL of a 0.5 mM DPPH solution in ethanol was added, and the absorbance was measured at 517 nm after 10 min. A control sample was prepared using 2.0 mL of ethanol and 0.5 mL of the same DPPH ethanolic solution, to check the radical stability. The percentage of radical scavenging activity (S) of each extract was calculated as S = 100 − [(A_*x*_/A_0_) × 100], where Ax is the absorbance of the DPPH solution in presence of the plant extract and A_0_ the absorbance of the control DPPH solution without the plant sample.

Total phenolic compounds (TPC) were quantified according to Blainski et al. (2013), by reaction with the Folin-Ciocalteu reagent. The methanol extracts were mixed with sodium bicarbonate and Folin-Ciocalteu reagent and left in the dark for 90 min. Absorbance was recorded at 765 nm, and the results expressed as equivalents of gallic acid (mg eq. GA g^−1^ DW).

Total “antioxidant flavonoids” (TF) were measured following the method described by Zhishen et al. (1999). The methanol extracts were mixed with NaNO_2_, followed by AlCl_3_ and NaOH, and the absorbance of the sample was measured at 510 nm. This protocol is often described to detect “total flavonoids” in the sample, although this is not strictly true. The method is based on the nitration of aromatic rings bearing a catechol group and only detects those phenolic compounds containing this chemical structure, which include several subclasses of flavonoids—such as flavonols or flavanols—but also other non-flavonoid phenolics, such as caffeic acid and derivatives. Nevertheless, the method was chosen since the metabolites determined by the reaction with AlCl_3_ are all antioxidants and there is a good correlation between their levels and the total antioxidant activity of the samples (Zhishen et al., 1999). To simplify, further on in the text we refer to the AlCl_3_-reactive compounds simply as “total flavonoids” (TF), and express their contents as equivalents of catechin (mg eq. C g^−1^ DW).

### Enzymatic antioxidant activities

#### Protein extraction and quantification

Crude protein extracts were prepared from plant material stored frozen at −80°C, following the procedure described in Gil et al. (2014). Protein concentration in the extracts was determined by the method of Bradford (1976), using the Bio-Rad reagent and bovine serum albumin (BSA) as standard.

#### Antioxidant enzyme activity assays

Catalase activity (CAT) was determined following the decrease in absorbance at 240 nm which accompanied the consumption of H_2_O_2_ (Δε = 39.4 mM^−1^ cm^−1^) upon the addition of the plant extracts (Aebi, 1984). One CAT unit was defined as the amount of enzyme that will decompose 1 μmol of H_2_O_2_ per min at 25°C. Glutathione reductase (GR) activity was quantified according to Connell and Mullet (1986), following the oxidation of NADPH, the cofactor in the GR-catalyzed reduction of oxidized glutathione (GSSG). One GR unit was defined as the amount of enzyme that will oxidise 1 μmol of NADPH per min at 25°C. Superoxide dismutase (SOD) activity was determined according to Beyer and Fridovich (1987) by monitoring the inhibition of nitroblue tetrazolium (NBT) photo-reduction, using riboflavin as the source of superoxide radicals. One SOD unit was defined as the amount of enzyme that causes 50% inhibition of NBT photo-reduction under the assay conditions. Minor modifications introduced in the aforementioned original assays are described in Gil et al. (2014).

### Data analysis

The location of the two species in the three salt marshes was established on the orthophotography Terrasit—72258-ecw provided by the “PNOA^©^, Instituto Geográfico Nacional de España - Institut Cartogràfic Valencià,” with the programme ArcGIS 2011.

Statistical analyses were performed using the program Statgraphics Centurion XVI. Before the analysis of variance, the Shapiro-Wilk test was used to check for validity of normality assumption and Levene test for the homogeneity of variance. If ANOVA requirements were accomplished, the significance of the differences among treatments was tested by one-way ANOVA at a 95% confidence level and *post-hoc* comparisons were made using the Tukey HSD test. Differences between the two species in each treatment were assessed by a *t*-test for the aforementioned confidence level.

All measured parameters in plants submitted to salt stress were correlated using principal component analysis (PCA), for 3 and 6 weeks in both *I. crithmoides* and *D. viscosa*. All means throughout the text include the SD.

## Results

### Field study

The distribution of the halophyte *Inula crithmoides* and the native-invasive *Dittrichia viscosa* was registered in three salt marshes in “La Albufera” Natural Park (Figure 1), and related to soil EC and moisture measurements in each point where the two species were present.

**Figure 2:**
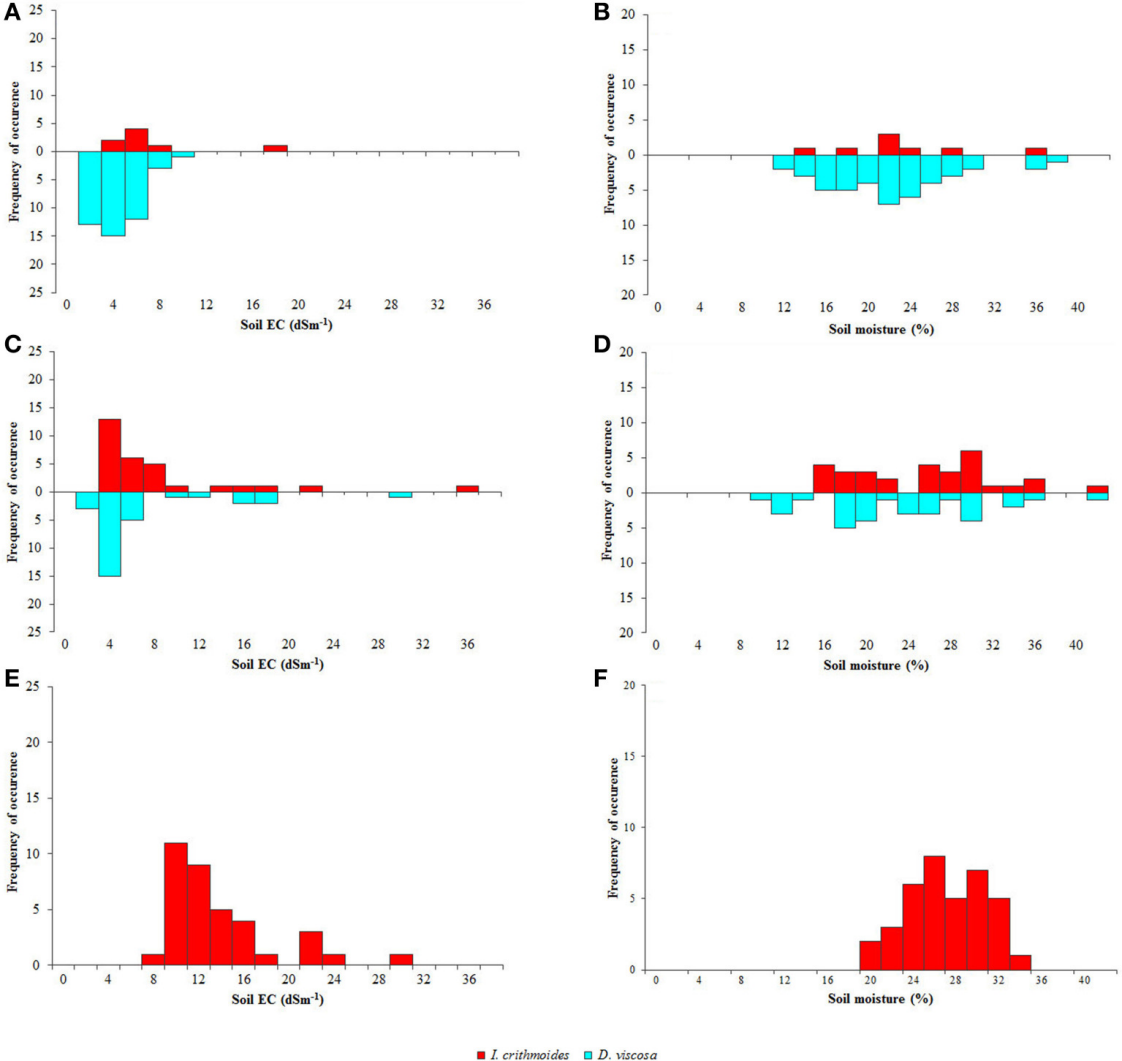
**Abundance and distribution of ***Dittrichia viscosa*** (light blue bars) and ***Inula crithmoides*** (red bars) plants in salt marshes 1 (A,B), 2 (C,D), and 3 (E,F), according to soil electric conductivity (EC, dS m^**−1**^) (A,C,E) and soil moisture (%) (B,D,F)**.

Figure 1A shows a general view of the area of the Natural Park where the field work was carried out, with the location of the three selected salt marshes. In the second row panels, the location points of the two species in the three salt marshes (Figures 1B1–B3, respectively) are represented. Soil electric conductivity and soil moisture values measured in each of those points are shown in Figure 2.

The first is the largest of the three analyzed salt marshes and has higher vegetation coverage. Although both species were present, *D. viscosa* was extremely abundant, contrasting with the scarcity of *I. crithmoides*. Soil salinity varied only slightly along the linear transect, except some occasional spots where electrical conductivity was higher. Mean values of EC ranged from 1 to 8 dS m^−1^, data that revealed relatively low salinity, optimal for *D. viscosa*, which invaded most of this salt marsh, as can be seen in Figure 1C1. Regarding soil moisture, *D. viscosa* was found in a range of 12–39%, but with an optimum at 20–24%. The few specimens of *I. crithmoides* were found at similar soil humidity, although not below 14%, and at salinities up to 16 dS m^−1^ (Figures 2A,B).

In the second salt marsh, both species were frequent but showed a different pattern of distribution: *I. crithmoide*s was present mostly in the central, more depressed part of the salt marsh, whereas *D. viscosa* was predominantly found on its borders. *I. crithmoides* grew over a wide range of salinity, from 4 to 36 dS m^−1^, whereas *D. viscosa* was concentrated at values of 1–6 dS m^−1^ and only a few individuals were localized at higher salinities, up to 20 dS m^−1^ (Figure 2C). Soil moisture values registered for the two species were similar, but *D. viscosa* was also found in drier locations (Figure 2D).

The third salt marsh is the smallest, with no vegetation in the centre but with high coverage on its border, including numerous specimens of *I. crithmoides*, but none of *D. viscosa*. This is related to its location, only a few meters from an effluent mouth, subjected to constant flooding during rainy periods. This implies that soil EC constantly changes and the salt marsh is wet most of the year, so that *I. crithmoides*, which apparently tolerates higher salinity, is favored. The species was present in soil areas with EC values up to 30 dS m^−1^, but more frequently at moderate salinities not exceeding 15 dS m^−1^ (Figure 2E). Soil moisture was higher than that registered in the other two salt marshes (Figure 2F).

### Plant growth inhibition under controlled stress conditions

**Figure 3:**
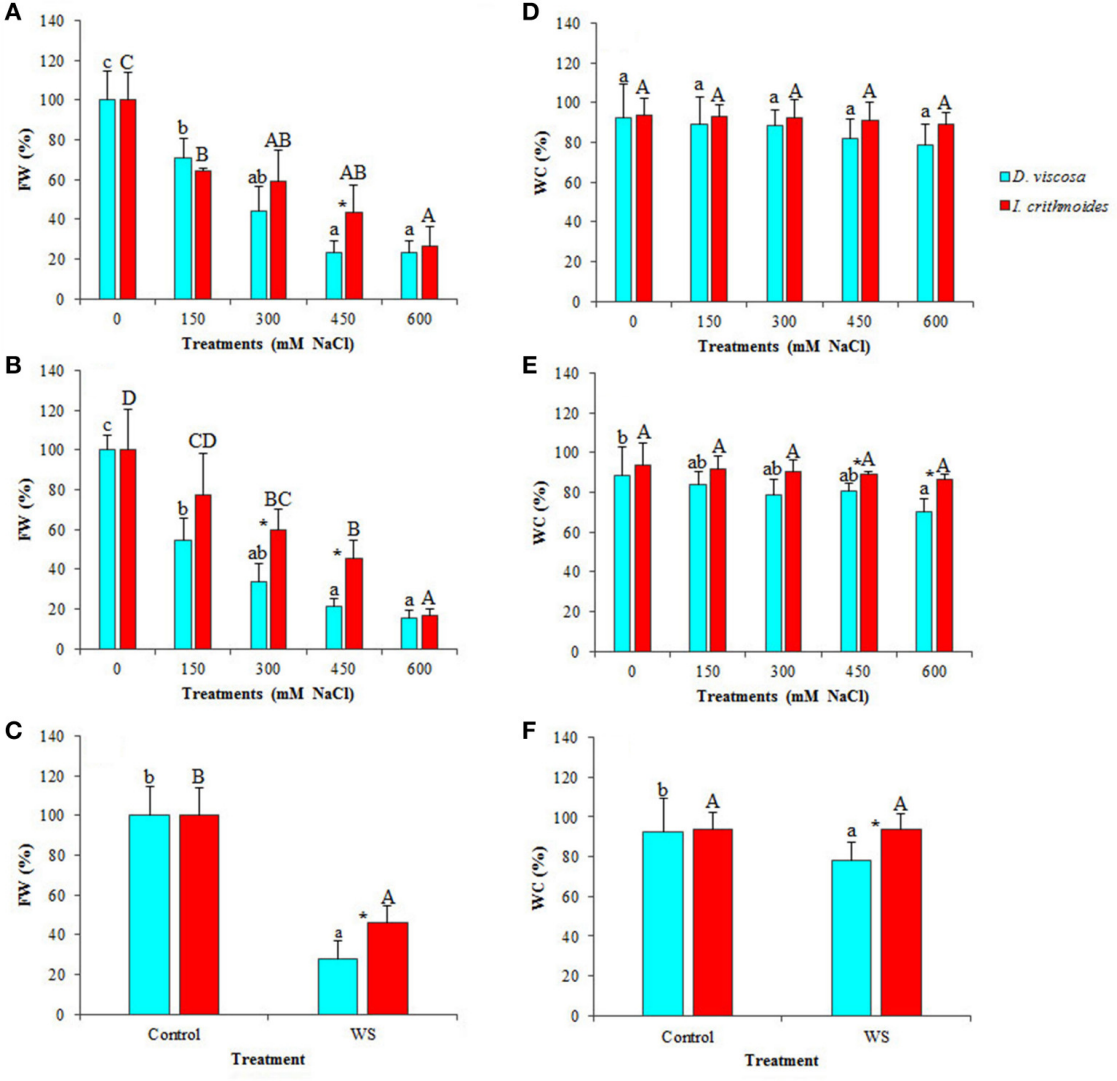
**Stress-induced growth inhibition in ***Dittrichia viscosa*** and ***Inula crithmoides*****. Leaf fresh weight [FW(%)] **(A–C)** and water content [WC(%)] **(D–F)** after 3 **(A,D)** and 6 **(B,E)** weeks of treatment with the indicated NaCl concentrations, or after 3 weeks of water stress **(C,F)**. For each treatment, FW is expressed as percentage of the absolute weight of the corresponding non-treated control, taken as 100%: 10.85 and 24.40 g for *D. viscosa* and 11.11 and 19.99 g for *I. crithmoides*, at 3 and 6 weeks of growth, respectively. Values shown are means ± SD (*n* = *5*). Different letters (lowercase for *D. viscosa* and capital letters for *I. crithmoides*) over the bars indicate significant differences between treatments for each species according to Tukey test (α = 0.05). Asterisks (^*^) indicate significant differences between the two species for the same treatment.

The leaf FW of both, *D. viscosa* and *I. crithmoides* salt-treated plants strongly decreased in parallel with increasing external NaCl concentrations, as compared to the corresponding non-treated controls. After 3 weeks of treatment, the relative FW reductions were slightly greater in *D. viscosa* than in *I. crithmoides*, but the differences were statistically significant only in the presence of 450 mM NaCl: 77% decrease in the former species, as compared to 57% in the latter (Figure 3A). When the salt treatment was prolonged for 6 weeks, these differences became clearer (Figure 3B), indicating that *I. crithmoides* is more resistant to salt stress than *D. viscosa*, in terms of inhibition of biomass accumulation. Similarly, after 3 weeks of water stress treatments—not all plants survived 6 weeks without watering—the relative FW reduction was again significantly higher in *D. viscosa* than in *I. crithmoides* (Figure 3C).

Both taxa showed a strong resistance to dehydration under stress conditions, indicating that the observed FW decrease was indeed due to stress-induced inhibition of growth and not simply to loss of water. In *I. crithmoides*, leaf water content did not vary significantly, as compared with the control plants, even after 6 weeks in the presence of 600 mM NaCl, the highest salt concentration tested, or after 3 weeks without watering (Figures 3D–F). In *D. viscosa*, a slight salt-induced decrease in the average leaf water content was detected, but the difference with the control was only significant after 6 weeks of treatment with 600 mM NaCl (Figure 3E); in water-stressed plants there was also a reduction in the mean leaf water content (ca. 15%) but, here again, the difference with the control plants was not statistically significant (Figure 3F). When comparing the two species, significant differences in water loss were detected in the 6-week treatment at high salt concentrations (450 and 600 mM NaCl; Figure 3E) and in the water stress treatment (Figure 3F); nevertheless, it should be pointed out that the differences observed between the two species, in absolute terms, were relatively small. Taken together, these results show that, although both taxa are quite resistant to stress, *I. crithmoides* is somewhat more tolerant than *D. viscosa* to salinity—in agreement with their relative distribution in the salt marshes—and also to drought.

### Photosynthetic pigments

Salt stress induced a relative reduction in the levels of photosynthetic pigments in the leaves of *D. viscosa* and *I. crithmoides* plants (Table 1), but its effects appeared to be weaker in the latter species, in agreement with its relatively higher salt tolerance. For example, in the 3 weeks treatment, chlorophyll a contents progressively decreased in *D. viscosa*, in parallel with increasing salt concentrations, down to about half of the control levels in the presence of 600 mM NaCl; in *I. crithmoides* plants, a similar reduction was observed under the same conditions, but lower salt concentrations had no significant effect (Table 1). After 6 weeks of salt treatment, the reduction in chlorophyll a levels was clearly stronger in *D. viscosa* (3.6-fold) than in *I. crithmoides* (twofold), and the same qualitative pattern was observed for chlorophyll b and total carotenoid contents (Table 1).

**Table 1:** **Photosynthetic pigments in leaves of salt-treated and water-stressed ***D. viscosa*** and ***I. crithmoides*** plants**.

**Photosynthetic pigment**	**Period of treatment (weeks)**	**Treatments (mM NaCl)**	**Species**		**Treatment**	**Species**	
			***D. viscosa***	***I. crithmoides***		***D. viscosa***	***I. crithmoides***
Chl a (mg g^−1^ DW)	3	0	28.33 ± 4.57c^*^	12.66 ± 1.64B^*^	Control	28.33 ± 4.57b^*^	12.66 ± 1.64B^*^
		150	18.43 ± 3.63ab^*^	13.04 ± 0.48B^*^	WS	16.84 ± 1.58a^*^	10.78 ± 1.99A^*^
		300	23.16 ± 2.50b^*^	13.22 ± 1.06B^*^			
		450	16.12 ± 2.508a^*^	11.64 ± 0.60B^*^			
		600	13.93 ± 2.23a^*^	6.56 ± 0.31A^*^			
	6	0	26.89 ± 4.33d^*^	13.11 ± 1.43C^*^			
		150	22.54 ± 1.55d^*^	11.17 ± 1.56C^*^			
		300	18.05 ± 1.25c^*^	5.83 ± 1.20AB^*^			
		450	15.76 ± 0.35b^*^	5.89 ± 0.44A^*^			
		600	7.52 ± 1.16a	6.62 ± 0.07B			
Chl b (mg g^−1^ DW)	3	0	13.77 ± 2.56c^*^	5.72 ± 0.70C^*^	Control	13.77 ± 2.56b^*^	5.72 ± 0.70B^*^
		150	5.68 ± 0.39a	5.22 ± 0.64*BC*	WS	5.62 ± 0.98a^*^	3.58 ± 0.69A^*^
		300	7.97 ± 0.98b^*^	5.15 ± 0.87BC^*^			
		450	6.74 ± 0.90ab^*^	4.43 ± 0.48B^*^			
		600	6.31 ± 1.49ab^*^	2.45 ± 0.53A^*^			
	6	0	12.07 ± 2.55d^*^	5.68 ± 0.57C^*^			
		150	9.33 ± 0.71d^*^	6.01 ± 0.98C			
		300	7.30 ± 0.28c^*^	2.77 ± 0.04A^*^			
		450	5.50 ± 0.26b^*^	2.91 ± 0.44A^*^			
		600	2.95 ± 0.34a^*^	3.90 ± 0.24B^*^			
Caro (mg g^−1^ DW)	3	0	4.02 ± 0.30d	3.19 ± 0.71C	Control	4.02 ± 0.30b	3.19 ± 0.71A
		150	3.39 ± 0.20c^*^	2.32 ± 0.21B^*^	WS	1.86 ± 0.37a	2.65 ± 0.41A
		300	2.74 ± 0.07b	2.40 ± 0.45BC			
		450	2.60 ± 0.18b^*^	1.98 ± 0.17B^*^			
		600	1.42 ± 0.28a	1.24 ± 0.13A			
	6	0	4.30 ± 0.29c^*^	3.30 ± 0.37D^*^			
		150	1.91 ± 0.29ab^*^	2.97 ± 0.35CD^*^			
		300	1.42 ± 0.36a^*^	2.70 ± 0.07C^*^			
		450	2.41 ± 0.26b	2.10 ± 0.05B			
		600	1.78 ± 0.43ab	1.65 ± 0.02A			

Chlorophyll a (Chl a), chlorophyll b (Chl b), and total carotenoids (Caro) contents, after 3-week and 6-week treatments with the indicated salt concentrations, and after 3 weeks of water stress. Values shown are means ± SD (n = 5). For each pigment, different letters (lowercase for D. viscosa and capital letters for I. crithmoides) in a column indicate significant differences between treatments according to Tukey test (α = 0.05). Asterisks (

* *) indicate significant differences between the two species for the same treatment. WS, water stress*.

Water stress also caused a reduction in the levels of photosynthetic pigments in both species, in relation to the non-stressed controls, and this decrease was again more pronounced in *D. viscosa* than in *I. crithmoides*. Thus, after 3 weeks without watering, chlorophyll a concentration in *D. viscosa* leaves was reduced by ca. 40% of the level in control plants, as compared to 15% in *I. crithmoides*, chlorophyll b by 60% of the control (vs. 37% in *I. crithmoides*), and total carotenoids by 54% (27% in *Inula*; Table 1).

### Osmolyte contents

**Figure 4:**
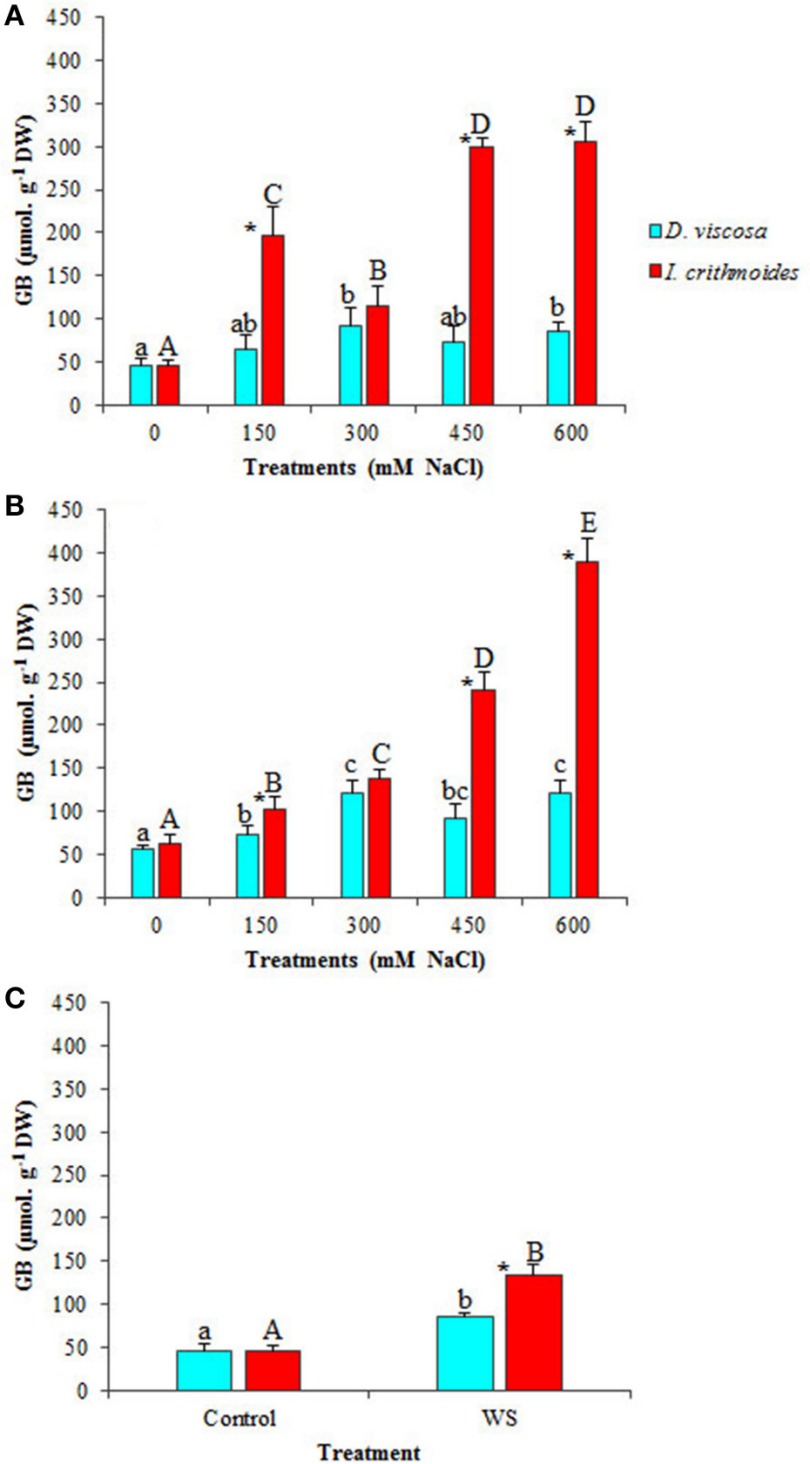
**Glycine betaine (GB) accumulation in leaves of ***D. viscosa*** and ***I. crithmoides*** stressed plants**. GB contents after 3 weeks **(A)** and 6 weeks **(B)** of treatment with the indicated NaCl concentrations, or after 3 weeks of water stress **(C)**. Values shown are means ± SD (*n* = *5*). Different letters (lowercase for *D. viscosa* and capital letters for *I. crithmoides*) over the bars indicate significant differences between treatments for each species according to Tukey test (α = 0.05). Asterisks (^*^) indicate significant differences between the two species for the same treatment.

Plants of the two analyzed species accumulated glycine betaine (GB) in their leaves, as a response to the treatment with increasing NaCl concentrations, but reaching much higher levels in *I. crithmoides* than in *D. viscosa* (Figures 4A,B). GB contents in control plants were similar in the two species, about 50 μmol g^−1^ DW; under the strongest salt stress conditions tested (6 weeks in the presence of 600 mM NaCl), GB levels increased about twofold in *D. viscosa*, but nearly eight-fold in *I. crithmoides* (Figure 4B). The high concentrations measured, almost 400 μmol g^−1^ DW, indicate that GB is the major functional osmolyte in *I. crithmoides*, responsible for osmotic adjustment in conditions of high soil salinity, as suggested by previous field studies (Gil et al., 2014).

GB contents did not show any significant change in *D. viscosa* plants after 3 weeks of water stress treatment; in *I. crithmoides*, on the contrary, water stress did induce the accumulation of this compound, albeit at much lower levels than those measured in salt-stressed plants, only a twofold increase over the control, approximately (Figure 4C).

**Figure 5:**
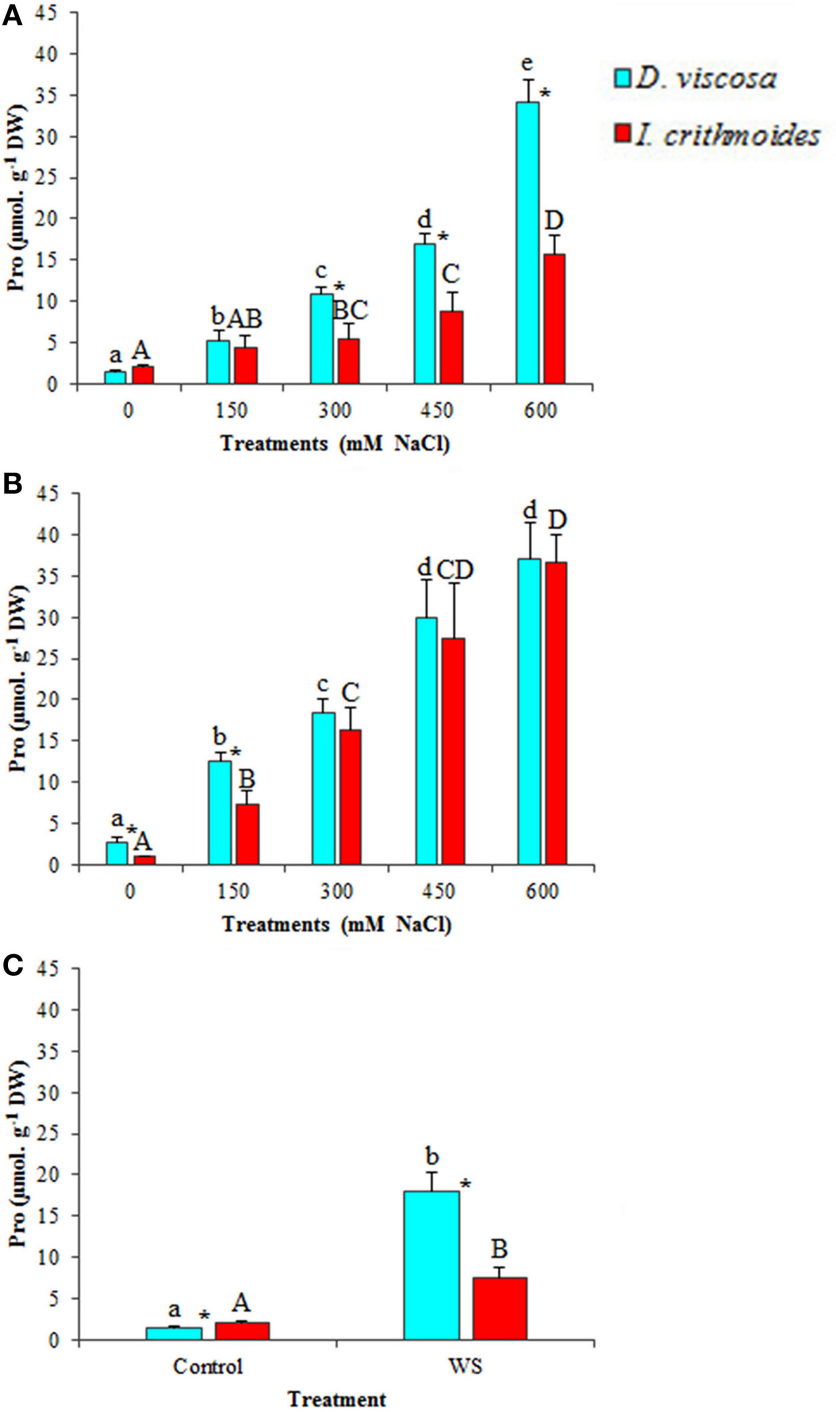
**Proline (Pro) accumulation in leaves of ***D. viscosa*** and ***I. crithmoides*** stressed plants**. Pro contents after 3 weeks **(A)** and 6 weeks **(B)** of treatment with the indicated NaCl concentrations, or after 3 weeks of water stress **(C)**. Values shown are means ± SD (*n* = *5*). Different letters (lowercase for *D. viscosa* and capital letters for *I. crithmoides*) over the bars indicate significant differences between treatments for each species according to Tukey test (α = 0.05). Asterisks (^*^) indicate significant differences between the two species for the same treatment.

Proline (Pro) was also measured in *D. viscosa* and *I. crithmoides* plants subjected to salt and water stress treatments (Figure 5). Pro levels augmented in both species upon treatment with NaCl, in a concentration-dependent manner. After 3 weeks in the presence of salt, Pro accumulation was relatively stronger in *D. viscosa*, reaching more than a 20-fold increase over control levels in the plants watered with 600 mM NaCl, as compared to ca. 7-fold in *I. crithmoides* under the same conditions (Figure 5A); when the treatment was prolonged to 6 weeks, however, *Inula* plants appeared to further increase Pro accumulation, so that its concentration was similar in the two species (Figure 5B). Water stress also led to Pro accumulation in the leaves of *D. viscosa* (12-fold increase over control values) and *I. crithmoides* (4-fold increase) plants (Figure 5C). Yet, it should be mentioned that the absolute Pro concentrations reached, always below 40 μmol g^−1^ DW, were one order of magnitude lower than those of GB; therefore, Pro could have, at best, a modest contribution to osmotic adjustment in the stressed plants.

Spectrophotometric determination of total soluble sugars, after reaction with phenol and sulfuric acid (Dubois et al., 1956), did not provide clear patterns of variation in response to salt of water stress (data not shown). Therefore, carbohydrates in the water-soluble fraction were separated, identified and quantified by HPLC. Only three major peaks were detected in the extracts of the two species, corresponding to arabinose (Ara), fructose (Fru), and glucose (Glu). These measurements revealed, as a general pattern, that the leaf contents of the three sugars increased in parallel with increasing external NaCl concentrations and with the time of treatment, for both species (Table 2). Thus, in *D. viscosa* plants grown in the presence of salt for 6 weeks, relative increases of ca. 1.5-, 2.8-, and 5-fold in the levels of Ara, Fru, and Glu, respectively, were observed when comparing the highest NaCl concentration tested (600 mM) and the non-stressed controls. Sugar contents in *I. crithmoides* control plants were in most cases lower than in *D. viscosa*, even not detectable in the case of Fru (after the 3-week growth period) and Glu (in samples collected at 3 and 6 weeks); however, the salt-induced increase in the levels of the three sugars was relatively larger in the former species, so that in the presence of 600 mM NaCl the contents of Ara, Fru, and Glu were significantly higher in *I. crithmoides* than in *D. viscosa* (Table 2). The absolute contents of the three sugars accumulated under the strongest salt stress conditions tested, taken together (100 μmol g^−1^ DW in *D. viscosa* and 160 μmol g^−1^ DW in *I. crithmoides*, approximately), could contribute significantly to osmotic adjustment, especially in *D. viscosa*, which accumulates much lower levels of GB.

**Table 2:** **Soluble sugars in leaves of salt-treated and water-stressed ***D. viscosa*** and ***I. crithmoides*** plants**.

**Soluble sugars**	**Period of treatment (weeks)**	**Treatments (mM NaCl)**	**Species**		**Treatment**	**Species**	
			***D. viscosa***	***I. crithmoides***		***D. viscosa***	***I. crithmoides***
Ara (μmol. g^−1^ DW)	3	0	16.71 ± 2.83a	13.96 ± 3.51*A*	Control	16.71 ± 2.83a	13.96 ± 3.51
		150	24.77 ± 2.48b^*^	45.84 ± 4.98B^*^	WS	45.71 ± 8.76b^*^	*N.D.*
		300	53.08 ± 8.19c	68.84 ± 5.51C			
		450	41.35 ± 3.71c	44.44 ± 5.22B			
		600	28.11 ± 5.95b^*^	75.27 ± 9.34C^*^			
	6	0	34.43 ± 4.36a	35.51 ± 6.27A			
		150	49.82 ± 6.94b	51.79 ± 6.87B			
		300	38.82 ± 7.06ab^*^	63.64 ± 7.51BC^*^			
		450	49.13 ± 5.51b^*^	69.54 ± 5.54C^*^			
		600	50.06 ± 8.42b^*^	72.55 ± 7.08C^*^			
Fru (μmol. g^−1^ DW)	3	0	3.11 ± 0.76a^*^	*N.D.*	Control	3.11 ± 0.76a^*^	*N.D.*
		150	5.65 ± 0.56b^*^	*N.D.*	WS	16.71 ± 2.90b^*^	*N.D.*
		300	7.51 ± 1.09c^*^	*N.D.*			
		450	9.88 ± 2.24c^*^	41.53 ± 4.50A^*^			
		600	7.68 ± 1.14c^*^	58.86 ± 3.78B^*^			
	6	0	7.22 ± 1.68a^*^	2.61 ± 0.56A^*^			
		150	12.07 ± 1.71b^*^	28.27 ± 3.41AB^*^			
		300	11.98 ± 1.61b^*^	37.00 ± 6.74B^*^			
		450	17.98 ± 1.13c^*^	56.62 ± 5.78C^*^			
		600	20.31 ± 1.72c^*^	51.25 ± 5.60C^*^			
Glu (μmol. g^−1^ DW)	3	0	1.07 ± 0.14a^*^	*N.D.*	Control	1.07 ± 0.14a^*^	*N.D.*
		150	14.05 ± 1.90b^*^	*N.D.*	WS	8.59 ± 0.55b^*^	*N.D.*
		300	14.32 ± 2.45b^*^	*N.D.*			
		450	24.28 ± 4.96c	23.06 ± 4.51*A*			
		600	30.75 ± 5.42c	31.65 ± 2.06*B*			
	6	0	5.33 ± 1.26a^*^	*N.D.*			
		150	27.13 ± 6.52b^*^	*N.D.*			
		300	18.27 ± 5.48b^*^	*N.D.*			
		450	25.31 ± 3.82b	29.85 ± 4.19A			
		600	28.01 ± 4.54b^*^	40.09 ± 7.01A^*^			

Arabinose (Ara), fructose (Fru), and glucose (Glu) contents, after 3-week, and 6-week treatments with the indicated salt concentrations, and 3 weeks of water stress. Carbohydrates were identified and quantified by HPLC of the water soluble leaf fraction. Values shown are means ± SD (n = 5). For each measured sugar, different letters (lowercase for D. viscosa and capital letters for I. crithmoides) in a column indicate significant differences between treatments according to Tukey test (α = 0.05). Asterisks (

* *) indicate significant differences between the two species for the same treatment. WS, water stress. (N.D. stands for “not detectable”)*.

An increase in sugar contents was also detected in *D. viscosa* plants, in response to water stress—ca. 2.7-fold (Ara), 5.4-fold (Fru), or 8-fold (Glu) higher than in the control plants. However, these sugars were not detected in the HPLC chromatograms of the leaf samples of *I. crithmoides* water-stressed plants (Table 2).

### Monovalent ions

Sodium (Na^+^), chloride (Cl^−^), and potassium (K^+^) contents were measured in leaves of *D. viscosa* and *I. crithmoides* plants, following the treatments with NaCl for 3 and 6 weeks (Table 3). Both species accumulated Na^+^ and Cl^−^ in response to salt stress, in a time and concentration-dependent manner; the relative increase in the concentration of these ions over the corresponding control plants, and the absolute levels reached, were higher in *I. crithmoides* than in *D. viscosa*. The measured K^+^ levels did not allow a clear correlation with the external salt concentrations and, interestingly, its patterns of variation differed in the two species. In *D. viscosa*, K^+^ contents, although fluctuating, showed a general tendency to decrease with increasing NaCl concentrations; in *I. crithmoides*, on the other hand, K^+^ concentration decreased at moderate salinity levels but increased again in the presence of high external salt concentrations (Table 3).

**Table 3:** **Monovalent ion contents in leaves of salt-treated ***D. viscosa*** and ***I. crithmoides*** plants**.

**Ions**	**Period of treatment (weeks)**	**Treatments (mM NaCl)**	**Species**	
		***D. viscosa***	***I. crithmoides***	
Na^+^(μmol g^−1^ DW)	3	0	144.84 ± 31.15*a*	216.05 ± 66.84*A*
		150	281.90 ± 87.77ab	382.23 ± 32.45B
		300	329.08 ± 58.52b^*^	865.37 ± 172.22C^*^
		450	502.13 ± 133.38c^*^	936.54 ± 151.86C^*^
		600	708.09 ± 217.07c^*^	1193.08 ± 259.86C^*^
	6	0	276.24 ± 92.40a	284.59 ± 98.03A
		150	989.88 ± 91.39b^*^	1503.95 ± 361.66B^*^
		300	1328.35 ± 278.56bc^*^	2234.02 ± 218.45C^*^
		450	1388.52 ± 164.95c^*^	2400.11 ± 260.86C^*^
		600	1474.03 ± 417.20c^*^	2441.94 ± 302.54C^*^
Cl^−^ (μmol g^−1^ DW)	3	0	100.72 ± 25.10*a*	145.06 ± 40.53A
		150	388.36 ± 80.85b	406.26 ± 14.34B
		300	421.25 ± 67.3b	540.19 ± 88.17C
		450	505.54 ± 3.12c^*^	1056.88 ± 185.94D^*^
		600	1008.65 ± 175.76d^*^	1353.29 ± 171.90D^*^
	6	0	122.00 ± 26.52a	139.16 ± 31.07A
		150	551.01 ± 17.23b^*^	1303.57 ± 50.60B^*^
		300	654.44 ± 158.87b^*^	1657.26 ± 49.86C^*^
		450	928.06 ± 59.83c^*^	1614.53 ± 87.60C^*^
		600	1020.33 ± 69.52c^*^	1752.25 ± 185.42C^*^
K^+^(μmol g^−1^ DW)	3	0	255.45 ± 38.88b^*^	346.25 ± 21.48B^*^
		150	205.15 ± 63.51ab	277.2 ± 52.33AB
		300	164.93 ± 9.47a^*^	209.95 ± 21.00A^*^
		450	232.17 ± 35.62b	308.33 ± 66.70B
		600	175.32 ± 48.53ab^*^	634.25 ± 43.98C^*^
	6	0	650.50 ± 64.32c^*^	546.53 ± 1.31D^*^
		150	347.58 ± 74.27b^*^	211.44 ± 22.94A^*^
		300	361.44 ± 42.00b^*^	264.90 ± 21.35B^*^
		450	361.44 ± 42.00b	443.12 ± 80.92C
		600	361.44 ± 42.00a^*^	435.70 ± 12.60C^*^

Sodium (Na^+^), chloride (Cl^−^), and potassium (K^+^) concentrations, after 3-week and 6-week treatments with the indicated salt concentrations. Values shown are means ± SD (n = 5). For each ion, different letters (lowercase for D. viscosa and capital letters for I. crithmoides) in a column indicate significant differences between treatments according to Tukey test (α = 0.05). Asterisks (

* *) indicate significant differences between the two species for the same treatment*.

Water stress did not induce any significant change in ion concentrations in the leaves of stressed plants of either species, in comparison to the non-stressed controls, as should be expected (data not shown).

### Oxidative stress responses

**Figure 6:**
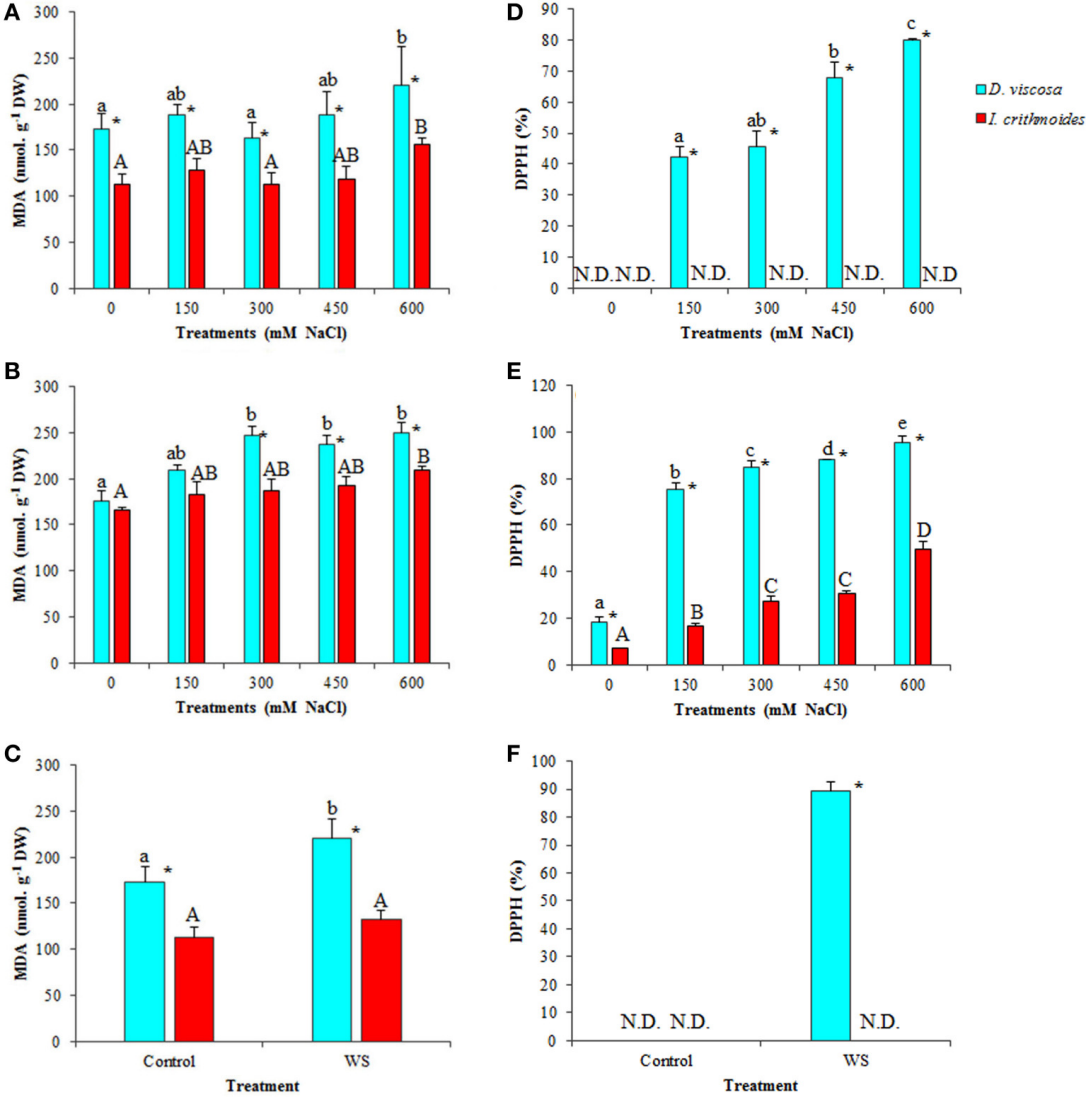
**Malondialdehyde (MDA) accumulation and DPPH scavenging activity (DPPH), in leaves of ***D. viscosa*** and ***I. crithmoides*** stressed plants**. MDA **(A–C)** and DPPH **(D–F)** after 3 weeks **(A,D)** and 6 weeks **(B,E)** of treatment with the indicated NaCl concentrations, or after 3 weeks of water stress **(C,F)**. Values shown are means ± SD (*n* = *5*). Different letters (lowercase for *D. viscosa* and capital letters for *I. crithmoides*) over the bars indicate significant differences between treatments for each species according to Tukey test (α = 0.05). Asterisks (^*^) indicate significant differences between the two species for the same treatment. (N.D. stands for “not detectable”).

Oxidative stress is usually associated to salt and water stress, through the generation of excess reactive oxygen species (ROS), toxic compounds that oxidize amino acid residues in proteins, unsaturated fatty acids in cell membranes, and DNA molecules, thus causing cellular damage (Halliwell, 2006). Malondialdehyde (MDA) is a product of membrane lipid peroxidation, considered an excellent marker of oxidative stress (Del Rio et al., 2005). MDA was found to increase moderately in plants of both species after 3 weeks of salt treatment, although significant differences with the corresponding controls were observed only in the presence of 600 mM NaCl (Figure 6A); prolonged treatments (6 weeks) led to a similar pattern of MDA accumulation in *I. crithmoides*, whereas significant differences with the control were already detected at 300 mM external NaCl concentration in *D. viscosa* plants (Figure 6B). Water stress also induced an increase of MDA levels in *D. viscosa*, but not in *I. crithmoides* (Figure 6C).

Absolute MDA contents were significantly lower in *I. crithmoides* than in *D. viscosa*, under all stress conditions tested (except for the 6-week treatment with 150 mM NaCl), although the differences between the two species were not extremely large (Figures 6A–C). These results indicate that drought and salinity cause a higher degree of oxidative stress in *D. viscosa* than in *I. crithmoides*, which is in agreement with the relative stress tolerance of the two taxa shown by the previous experiments and by their distribution in nature.

Scavenging activity of the DPPH radical—used to estimate total antioxidant activity—was not detected in *I. crithmoides* leaf extracts, neither of control plants, nor in water-stressed or salt-stressed plants after the 3-week treatments (Figures 6D,F). A concentration-dependent increase of activity was however observed in plants treated with increasing salt concentrations for 6 weeks, reaching 45% in the presence of 600 mM NaCl (Figure 6E). In accordance with the relatively higher degree of oxidative stress affecting *D. viscosa*, significant concentration and time-dependent increases in antioxidant activity were detected in plants of this species, reaching 90% upon application of water stress (Figure 6F), 80% in the presence of 600 mM NaCl for 3 weeks (Figure 6D) or up to 95% in the 6-week salt treatment (Figure 6E).

**Figure 7:**
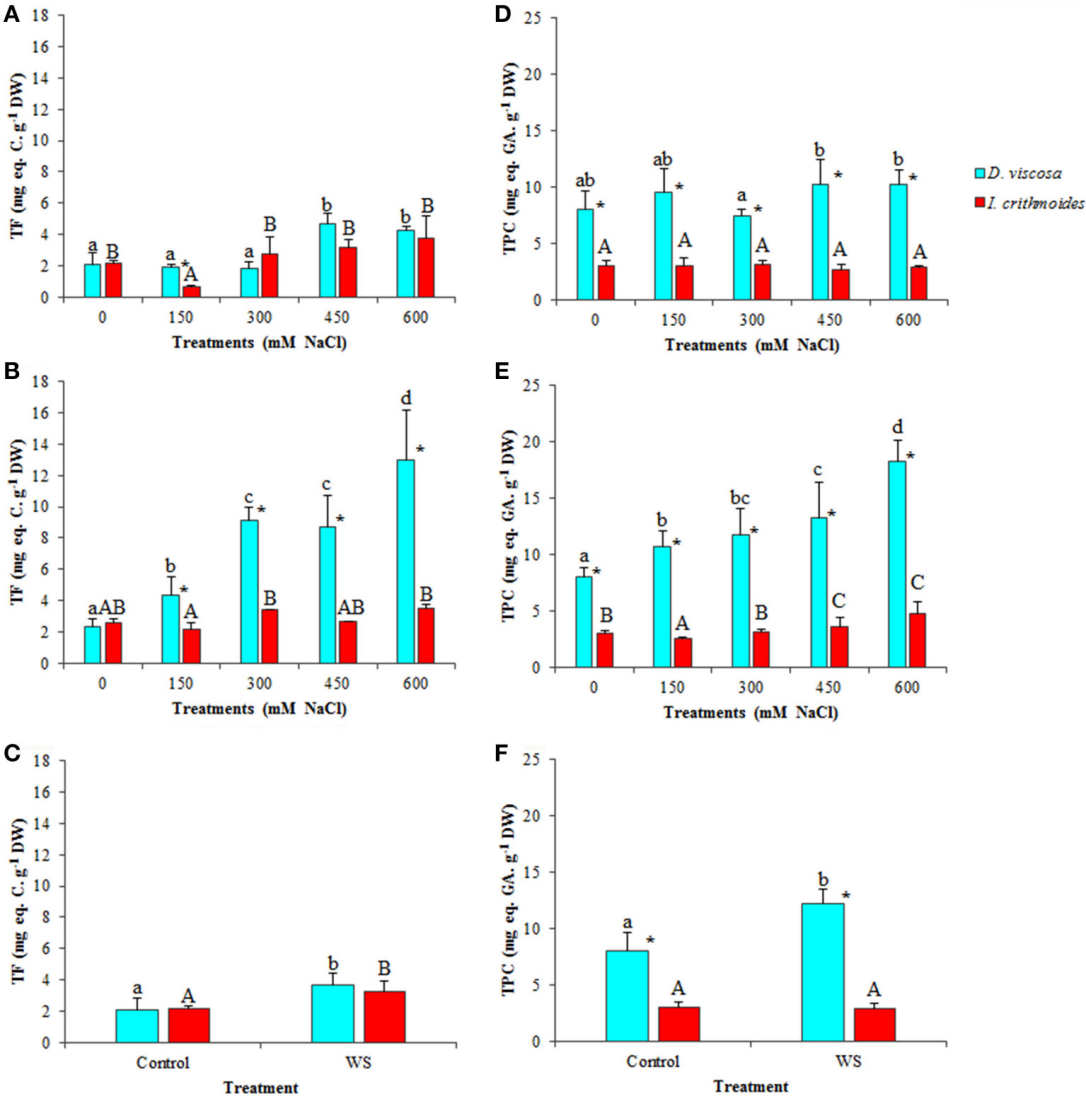
**Total flavonoids (TF) and total phenolic compounds (TPC) accumulation in leaves of ***D. viscosa*** and ***I. crithmoides*** stressed plants**. TF **(A–C)** and TPC **(D–F)** contents after 3 weeks **(A,D)** and 6 weeks **(B,E)** of treatment with the indicated NaCl concentrations, or after 3 weeks of water stress **(C,F)**. Values shown are means ± SD (*n* = *5*). Different letters (lowercase for *D. viscosa* and capital letters for *I. crithmoides*) over the bars indicate significant differences between treatments for each species according to Tukey test (α = 0.05). Asterisks (^*^) indicate significant differences between the two species for the same treatment.

Phenolic compounds and particularly flavonoids possess well-established antioxidant and ROS scavenging activities, and can be considered as good examples of non-enzymatic antioxidant metabolites induced in plants as a response to abiotic stress conditions causing secondary oxidative stress. Total flavonoid (TF) contents increased in salt-stressed plants of *D. viscosa*, an effect that was more clearly observed after 6 weeks of treatment with salt, reaching a 6-fold increase in the presence of 600 mM NaCl, over the level in non-treated plants (Figures 7A,B). In *I. crithmoides*, TF levels were lower than those measured in *D. viscosa* under most tested conditions, and their variations with increasing salinity were also smaller and, generally, not statistically significant (Figures 7A,B). Water stress induced a slight (less than twofold) increase in TF contents in both species, but no significant differences were observed between *I. crithmoides* and *D. viscosa* plants (Figure 7C).

The patterns of variation of total phenolic compounds (TPC), in response to increasing salt concentration, were similar to those of TF, although their absolute levels were clearly higher in *D. viscosa* than in *I. crithmoides* under all conditions tested. No significant changes were observed, in general, after 3 weeks of salt treatments (Figure 7D), while in the 6-week treated plants TPC accumulated with increasing NaCl concentrations in both species, although the relative increase over the corresponding control was more pronounced in *D. viscosa* than in *I. crithmoides* (Figures 7D,E). Regarding water-stressed plants, a significant increase of TPC levels was detected in *D. viscosa* but not in *I. crithmoides* (Figure 7F).

The specific activity of several antioxidant enzymes was determined in leaf protein extracts prepared from plants of the two investigated species. Although some changes were observed in response to the applied salt and water stress treatments, in general the differences were small and, in most cases, statistically non-significant (Table 4). Thus, in *I. crithmoides*, catalase (CAT) activity did not vary in the presence of salt, under all conditions tested. The specific activities of glutathione reductase (GR) fluctuated in response to increasing salt concentrations, but the differences between the control plants and those watered with 600 mM NaCl were again non-significant. Only superoxide dismutase (SOD) activity increased significantly, albeit slightly (up to 1.5-fold), in the 6-week treatment with high NaCl concentrations. Regarding the *I. crithmoides* water-stressed plants, no significant changes were detected in any of the antioxidant activities tested (Table 4). In *D. viscosa*, apart from a small decrease in CAT activity in the presence of 600 mM NaCl for 6 weeks, the only remarkable change was the activation of SOD (up to about 3.6-fold) induced by increasing salt concentrations. Contrary to *I. crithmoides*, water stress led to significant (but again small) variations of antioxidant enzyme activities in *D. viscosa*, reducing CAT and GR, and increasing SOD, as compared to the non-stressed controls (Table 4).

**Table 4:** **Antioxidant enzyme activities in leaves of salt-treated and water-stressed ***D. viscosa*** and ***I. crithmoides*** plants**.

**Antioxidant enzyme**	**Period of treatment (weeks)**	**Treatments (mM NaCl)**	**Species**		**Treatment**	**Species**	
			***D. viscosa***	***I. crithmoides***		***D. viscosa***	***I. crithmoides***
CAT (U mg^−1^ protein)	3	0	16.24 ± 1.36a	13.70 ± 3.48A	Control	16.24 ± 1.36b	13.70 ± 3.48A
		150	14.64 ± 0.99a	15.77 ± 1.22A	WS	11.22 ± 2.10a	12.96 ± 1.56A
		300	17.91 ± 2.67a	13.64 ± 1.71A			
		450	13.74 ± 1.58a	14.02 ± 2.67A			
		600	12.52 ± 2.40a	13.34 ± 0.84A			
	6	0	15.35 ± 1.74b	14.32 ± 2.14A			
		150	16.11 ± 2.13b	15.73 ± 1.09A			
		300	13.07 ± 1.87b	15.06 ± 1.67A			
		450	12.29 ± 0.77b	14.94 ± 2.40A			
		600	9.02 ± 1.30a^*^	13.80 ± 1.63A^*^			
GR (U mg^−1^ protein)	3	0	0.05 ± 0.00c^*^	0.11 ± 0.01A^*^	Control	0.05 ± 0.00b^*^	0.11 ± 0.01A^*^
		150	0.16 ± 0.02d^*^	0.24 ± 0.03B^*^	WS	0.04 ± 0.00a^*^	0.09 ± 0.01A^*^
		300	0.06 ± 0.00c^*^	0.15 ± 0.02AB^*^			
		450	0.03 ± 0.00a^*^	0.23 ± 0.04B^*^			
		600	0.04 ± 0.00b^*^	0.11 ± 0.02A^*^			
	6	0	0.06 ± 0.01a	0.09 ± 0.02AB			
		150	0.08 ± 0.01ab^*^	0.12 ± 0.02BC^*^			
		300	0.09 ± 0.02ab	0.07 ± 0.01A			
		450	0.1 ± 0.02b^*^	0.16 ± 0.03C^*^			
		600	0.06 ± 0.01a^*^	0.11 ± 0.02B^*^			
SOD (U mg^−1^ protein)	3	0	21.24 ± 4.56a	18.92 ± 3.32A	Control	21.24 ± 4.56a	18.92 ± 3.32A
		150	20.04 ± 3.08a	22.24 ± 5.16A	WS	39.04 ± 5.24b^*^	25.08 ± 5.48A^*^
		300	38.16 ± 3.68b^*^	19.24 ± 2.16A^*^			
		450	37.92 ± 2.12b^*^	18.92 ± 3.32A^*^			
		600	48.92 ± 5.44c^*^	21.00 ± 2.84A^*^			
	6	0	25.68 ± 4.12a	26.64 ± 2.48A			
		150	44.04 ± 6.68b^*^	24.68 ± 3.48A^*^			
		300	49.96 ± 6.84b^*^	27.12 ± 3.52A^*^			
		450	68.60 ± 8.48c^*^	32.32 ± 2.76B^*^			
		600	93.40 ± 9.80d^*^	38.04 ± 4.44B^*^			

Catalase (CAT), glutathione reductase (GR), and superoxide dismutase (SOD) specific activity, after 3-week and 6-week treatments with the indicated salt concentrations, and 3 weeks of water stress. Values shown are means ± SD (n = 5). For each enzyme, different letters (lowercase for D. viscosa and capital letters for I. crithmoides) in a column indicate significant differences between treatments according to Tukey test (α = 0.05). Asterisks (^*^) indicate significant differences between the two species for the same treatment. WS, water stress.

### Principal component analyses

**Figure 8:**
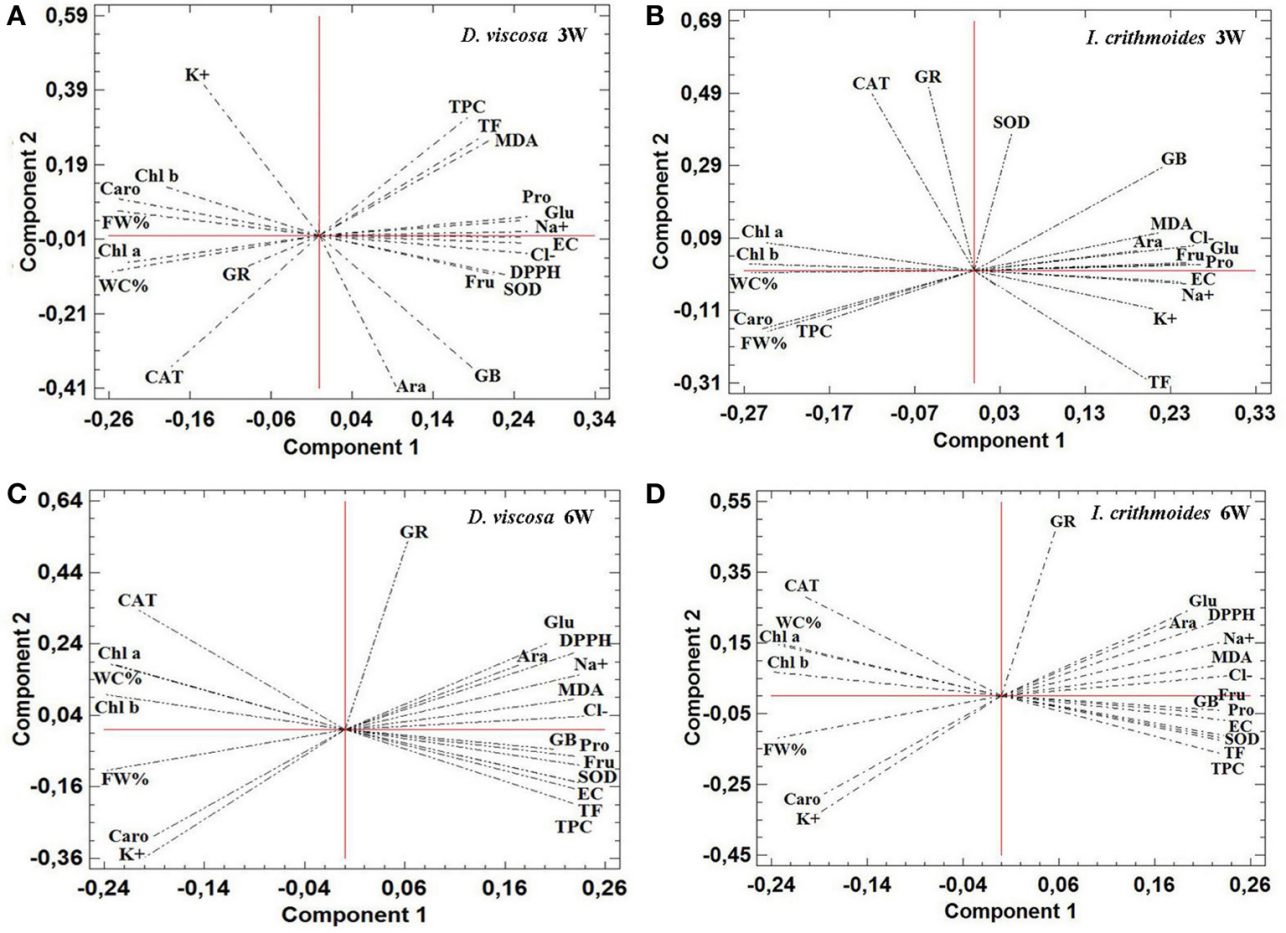
**Principal Component Analysis (PCA)**. Site score plots of the studied variables on the two principal components (1 and 2) for *D. viscosa* **(A,C)** and *I. crithmoides* **(B,D)** plants, after 3 weeks **(A,B)** and 6 weeks **(C,D)** of salt treatments. PCAs included, as the analyzed variables: electrical conductivity of the substrate at the end of the salt treatments (EC), fresh weight percentage (FW%), water content percentage (WC%), chlorophyll a (Chl a), chlorophyll b (Chl b), total carotenoids (Caro), sodium ions (Na^+^), potassium ions (K^+^), chloride ions (Cl^−^), glycine-betaine (GB), proline (Pro), arabinose (Ara), fructose (Fru), glucose (Glu), malondialdehyde (MDA), total antioxidant activity—DPPH scavenging activity—(DPPH), total flavonoids (TF), total phenolic compounds (TPC), and specific activity of catalase (CAT), glutathione reductase (GR), and superoxide dismutase (SOD). Values of the different parameters were obtained from the experiments shown in Figures 3–6 and Tables 1–4, and in “Supplementary Material” Table S1.

Principal component analyses (PCAs) were performed independently for *D. viscosa* and *I. crithmoides*, and for the 3-week and 6-week salt treatments, and included all measured parameters (Figure 8). In the four PCAs shown, two components with an Eigenvalue equal to or greater than 1 explained a cumulative percentage of variance of more than 80%. The first component (X-axis) was determined by the electrical conductivity of the substrates (shown as Supplementary Material in Table S1), and was found to be strongly correlated, negatively, with growth parameters (FW%, WC%) and contents of photosynthetic pigments (Chl a, Chl b, and Caro), and positively with toxic ions (Na^+^ and Cl^−^) levels, osmolyte (GB, Pro, Glu, Fru, Ara) contents, MDA, total antioxidant activity (DPPH), non-enzymatic antioxidants (TPC and TF), or SOD activity. Although similar correlation patterns were observed for each species, regardless of the duration of the treatment, the variation explained by the first component increased from about 67% at 3 weeks to 79% at 6 weeks, in both *D. viscosa* and *I. crithmoides*. This is in agreement with the fact that, in general, the loading vectors of the analyzed variables presented smaller angles with the X-axis—that is, higher correlation with salinity—in the PCAs corresponding the 6 weeks of treatment, due to the prolonged salt stress effects. The joint analysis of all variables indicated that the responses of the two species to salt stress are the same or very similar, qualitatively, and that quantitative differences are most clearly manifested after a longer treatment.

## Discussion

Salt marsh ecosystems are characterized by quick changes in their environmental conditions (Chapman, 1974). In Mediterranean salt marshes, spatial and temporal gradients of salinity and soil moisture have been reported among the most important physical factors for plant distribution (Álvarez Rogel et al., 2001). A strong seasonal variation in soil electric conductivity was reported by Gil et al. (2011, 2014) in the area of study, which is explained by high evapotranspiration in summer, causing an upward movement of water with dissolved salts that accumulate in the soil upper layers, thus increasing its salinity. Therefore, to be able to compare the field data sets, soil EC and moisture were analyzed in the three salt marshes at approximately the same time, in early spring, when soil humidity was sufficient to perform direct *in situ* measurements with a portable sensor.

*Dittrichia viscosa* is a species adapted to a wide range of environmental stresses (Parolin et al., 2014), and is frequent in many different habitats, especially in those with anthropic influence. It has also been reported from salt meadows and saline tamarisk thickets (Korakis and Gerasimidis, 2006), and seems to tolerate relatively high concentrations of NaCl in artificial experimental conditions, yet it is not considered as a true halophyte (Curadi et al., 2005; Maciá-Vicente et al., 2012). When comparing the distribution of the two species in the selected salt marshes, soil EC appeared as the major restrictive ecological factor for *D. viscosa*. At low and moderate salinities, as in the first salt marsh, it was by far more abundant than *I. crithmoides*, and practically invaded the whole area. In the second salt marsh, where both species were present, *D. viscosa* was localized mostly on the edges, at lower soil EC, while in the central and more depressed zone, with higher salinity, *I. crithmoides* was more abundant. This latter species was the only one of the two growing in the third, more humid and saline salt marsh.

The simplest interpretation of the field data would be to assume that *I. crithmoides* is an “obligate” halophyte, which requires higher salt levels than *D. viscosa* in its natural environment. However, we consider “obligate” as a misleading term, as it may suggest that the plants necessarily require salt for optimal growth. In fact, *I. crithmoides* plants grew better in the absence than in the presence of NaCl, as shown here and has also been recently reported by Pardo-Domènech et al. (2015). Moreover, when comparing *I. crithmoides* cultivated on pots with different substrates, optimal growth was found on salt-free and nutrient-rich substrates, such as peat and garden soil, and not on soil sampled in the salt marsh where seeds were collected (Grigore et al., 2012). These findings indicate that in non-saline or low to moderate salinity areas *I. crithmoides* is outcompeted by other species, such as *D. viscosa*; only at higher salinities, the species becomes truly competitive with respect to *D. viscosa*, which is less salt tolerant.

It is generally accepted that plant tolerance to abiotic stresses, including drought and salinity, is mostly dependent on the activation of a series of conserved response mechanisms, such as the control of ion homeostasis and the accumulation of specific osmolytes to ensure cellular osmotic balance, or the activation of antioxidant systems to counteract oxidative stress which occurs in such stressful conditions (Flowers et al., 1986; Hare et al., 1998; Zhu, 2001; Flowers and Colmer, 2008). These basic mechanisms are not specific for stress tolerant species, but shared by all plants, and the wide range of tolerance in different species is mostly attributed to the relative efficiency of those mechanisms; in other words, to quantitative rather than qualitative differences in the responses to stress. Studies on related taxa with different degrees of tolerance to stress could be extremely useful for a better understanding of the relative contribution of different stress responses to stress tolerance in a given species or group of related taxa (Boscaiu et al., 2013).

*D. viscosa* and *I. crithmoides* are genetically related, since they belong to taxonomically close genera—*D. viscosa* was formerly included in the *Inula* genus, as *I. viscosa*—although they show different ecological behavior. Therefore, according to the aforementioned ideas, the same mechanisms of tolerance should operate in both species, albeit with different efficiency. All our results indicate that this is, indeed, the case: although quantitative differences were noticed, *D. viscosa* and *I. crithmoides* showed the same patterns of growth inhibition under salt and water stress conditions, and the physiological and biochemical responses to stress were similar in the two species, as discussed below.

Apart from growth inhibition, leaf photosynthetic pigments appear to be reliable abiotic stress markers, since reductions in chlorophylls (a and b), and carotenoid contents have been reported to correlate closely with the degree of salinity or drought affecting plants of several species (e.g., Sairam et al., 2002; Jaleel et al., 2009; Hernández et al., 2015; Schiop et al., 2015). In our experiments, a decrease in the levels of these compounds was registered in all stress treatments in the two species, but the reduction was more pronounced in *D. viscosa*, which is clearly more affected by salt and water stress than *I. crithmoides*.

A general response to salt stress in plants, which may contribute to tolerance, is based on the control of ion transport. Accumulation of inorganic ions in the aerial part of the plants is an advantageous mechanism to increase osmotic pressure, more economical in terms of energy consumption than only the synthesis of organic solutes for osmotic adjustment (Raven, 1985). Toxic Na^+^ and Cl^−^ ions are maintained at low cytosolic concentrations through ion sequestration in vacuoles by selective uptake, according to the widely accepted “ion compartmentalization hypothesis” (Flowers et al., 1986; Glenn et al., 1999). This strategy is used preferentially by glycophytes (within their limits of resistance) and dicotyledonous halophytes, whereas salt-tolerant monocots cope with high salinity in the soil mostly by limiting Na^+^ transport to the leaves. Both, *I. crithmoides* and *D. viscosa* plants accumulated Na^+^ (and Cl^−^) in their leaves, in response to increasing NaCl concentrations in the pots, although significantly higher levels were measured in *I. crithmoides*; this species is succulent, which facilitates the accumulation of ions in the vacuoles. These results point to a higher efficiency in the mechanism of transport of toxic ions from the roots to the leaves, which would be extremely meaningful in conditions of high salinity, conferring *I. crithmoides* a clear advantage over *D. viscosa* in colonizing saline environments.

The accumulation of sodium is generally associated with a decrease of potassium levels in plants, mostly due to the competition for the same binding sites. Na^+^ interferes with K^+^ transport by using its physiological transport systems (Greenway and Munns, 1980; Flowers et al., 1986) and by inducing a depolarization of the plasma membrane, triggering the activation of outward-rectifying K^+^ channels and consequently the loss of K^+^ (Shabala et al., 2003, 2005). Maintaining a relatively high cellular K^+^ level under salt stress is another fundamental mechanism of tolerance, described in some halophytes, such as *Thellungiella halophila*, a salt-tolerant relative of the glycophyte *Arabidopsis thaliana* (Volkov et al., 2003). Decreasing K^+^ levels with increasing external Na^+^ concentrations were detected in *D. viscosa*, especially after 6 weeks of salt treatments, but it is particularly interesting to note the significant *increase* of K^+^ contents observed in the more salt-resistant *I. crithmoides* at high (450–600 mM) NaCl concentrations—under lower salinity conditions, a decrease in K^+^ was also detected in this species, as expected—suggesting the activation of potassium transport to the leaves. This could partly compensate the accumulation of sodium, thus contributing to salt tolerance in *I. crithmoides* by avoiding a drastic reduction of K^+^/Na^+^ ratios.

Accumulation of high concentrations of toxic ions in the vacuole requires the synthesis of compatible solutes in the cytoplasm, to maintain osmotic balance. The higher tolerance to stress of *I. crithmoides*, as compared to *D. viscosa*, appear to be partly dependent on the accumulation of higher levels of specific osmolytes, providing better osmotic adjustment under stress. Glycine betaine has been reported as the major osmolyte in *I. crithmoides*, accumulating at very high concentrations in response to controlled salt treatments in the laboratory (Pardo-Domènech et al., 2015), and also under stressful environmental conditions in the field (Gil et al., 2014). To our knowledge, up to now there are no published reports on the mechanisms of response to abiotic stress in *D. viscosa*. In the present study, we measured similar levels of GB in control plants of *D. viscosa* and *I. crithmoides* and observed significant increases in response to the salt treatments, as well as—to a lesser extent—in response to water stress. Yet, under the same conditions, GB accumulation was stronger in *I. crithmoides* than in *D. viscosa*. Salt-induced accumulation of some specific soluble sugars, namely Ara, Fru, and Glu, was also detected in the two species, reaching levels that would significantly contribute to cellular osmotic adjustment—especially in *D. viscosa*, due to its weaker accumulation of GB. Here again, the relative increases over the control plants and the absolute contents of the three sugars measured in the presence of high salt concentrations were bigger in the more salt tolerant *I. crithmoides*. Interestingly, these sugars do not appear to be involved in the responses to drought of the latter species, whereas their levels increase significantly in water-stressed *D. viscosa* plants. The possible role of soluble sugars in the mechanisms of abiotic stress tolerance in plants is often difficult to assess, due to their multiple additional functions in the cell as major energy sources, precursors of metabolic compounds and signaling molecules. Nevertheless, there is still much evidence for the contribution of soluble carbohydrates to salt and drought tolerance (reviewed in Gil et al., 2013), as we have observed in the two species investigated here.

Proline is one of the commonest osmolytes in plants, and is synthesized in response to many different stressful conditions, such as salinity, drought, cold, high temperature, nutritional deficiencies, heavy metals, air pollution, or high UV radiation (Hare and Cress, 1997; Grigore et al., 2011; Boscaiu et al., 2013). It is well known that Pro levels increase in conditions of abiotic stress in many plant species, therefore Pro accumulation is a general response to stress; yet—as for other osmolytes—this does not mean that Pro is necessarily involved in stress tolerance mechanisms, as not always higher Pro levels correlate with increased tolerance (e.g., Lutts et al., 1996; Ashraf and Foolad, 2007; Chen et al., 2007). This seems to be the case in our experiments, since *D. viscosa* showed accumulation of Pro to higher levels than the more tolerant *I. crithmoides*. In any case, Pro contents, even under the strongest stress conditions tested, were too low to contribute significantly to osmotic adjustment in either species, thus ruling out a direct role of this compound in stress tolerance in the investigated taxa.

Different abiotic stresses, including salinity and drought, cause oxidative stress in plants as a secondary effect, by inducing a large increase in the amount of reactive oxygen species (ROS) (Van Breusegem and Dat, 2006). When in excess, ROS cause cellular damage by oxidising proteins, membrane lipids and DNA (Apel and Hirt, 2004; Halliwell, 2006). Consequently, another general response to abiotic stress in plants is based on the activation of enzymatic and non-enzymatic antioxidant systems, the latter including many flavonoids and other phenolic compounds. There is overwhelming evidence that these secondary metabolites participate in the responses of plants to practically all types of abiotic stress (Winkel-Shirley, 2002; Treutter, 2005, 2006; Gould and Lister, 2006; Pollastri and Tattini, 2011), due to their strong antioxidant character and ROS scavenging activity.

In agreement with its higher tolerance, under the same stressful conditions *I. crithmoides* showed lower levels of oxidative stress than *D. viscosa*—as established from measurements of MDA contents in the plants—both in the presence of increasing NaCl concentrations, and after the water stress treatment. Since *D. viscosa* is relatively more affected by oxidative stress, it should be expected that these plants need to activate stronger antioxidant systems than *I. crithmoides* as a defense mechanism against salt or water stress. This is indeed the case, at least regarding measurements of total antioxidant activity in the plant extracts—by the DPPH radical scavenging assay—accumulation of antioxidant secondary metabolites such as total phenolic compounds and flavonoids, and the specific activity of the antioxidant enzyme superoxide dismutase (SOD), all of which showed significantly higher values in *D. viscosa* than in *I. crithmoides* under all tested conditions. These results partly confirm previous (field) data from our laboratory, indicating that in their natural habitat highly salt-tolerant species, including *I. crithmoides*, use very efficient mechanisms of response to salinity—based on the control of ion transport and the accumulation of specific osmolytes—thus avoiding the generation of oxidative stress and the need to activate antioxidant systems (Gil et al., 2014; Bautista et al., 2016).

Summarizing, determination of growth parameters and several biochemical stress markers in *I. crithmoides* and *D. viscosa* plants subjected to controlled salt and water stress treatments, the results of individual experiments and the joint analysis of all variables by PCA, indicated that these two genetically related species use the same physiological mechanisms to respond to stress. These mechanisms are mostly based on the transport of toxic ions from the roots to the leaves—where they are presumably sequestered in vacuoles—and the accumulation of specific osmolytes (GB and the sugars Ara, Fru, and Glu) for osmotic adjustment. Differences between the two species are quantitative: ion transport and compartmentalization in vacuoles is more efficient *I. crithmoides* than in *D. viscosa*, and the osmolytes (especially GB) accumulate at higher levels in the former species, thus explaining the (slightly) higher stress tolerance of *I. crithmoides*. The possible activation of K^+^ transport to the leaves under strong salinity conditions may also contribute to the higher salt tolerance in this species. The results obtained in controlled experiments in the greenhouse corresponded to the relative distribution of the two species in nature, as only *I. crithmoides* was found in areas with the highest salinity. Still, *D. viscosa* proved to be quite resistant to salt stress, tolerating low and moderate salinities in the field.

Coming back to the main issue of this paper, whether *D. viscosa* may be a threat for Mediterranean salt marsh species, the answer is negative, if we refer only to true halophytes growing in areas with high soil salinity—such as *I. crithmoides*—because its mechanisms of adaptation to salinity are less efficient. Yet *D. viscosa* can endanger less competitive taxa, since it is quite resistant to lower salinities. From an ecological point of view, the lower salinity makes *D. viscosa* more competitive, as it characterizes an advanced stage of anthropically influenced or degraded communities (*Inulo viscosae-Oryzopsietum miliaceae*). *I. crithmoides* is a characteristic species from salt marsh communities (*Inulo crithmoidis*-*Tamaricetum boveanae*) with possible sub-nitrophillous behaviour, so it coexists in the salt marshes with *D. viscosa*, but cannot leave the salt marsh. The consequence is that, although *D. viscosa* could not directly cause the disappearance of *I. crithmoides*, it is a powerful chamaephyte from a serial scrub that will be stronger in this habitat when suffering land degradation. However, salt marshes in the area of study shelter only a few rare halophyte species, including several endemics of the genus *Limonium*. Much more diverse flora, with a higher rate of endemicity, is located on the borders of the salt marshes, where *D. viscosa* is extremely competitive. Therefore, management programs of protected areas in the region should include not only the control of alien invasive species, but also the native-invasive *D. viscosa*. Moreover, the invasive character of *D. viscosa* in disturbed environments should be taken into account when using this species to restore degraded and polluted areas, or as an intermediate species after invasions by exotic taxa. In relation to habitat preservation, measures should not go only in the direction of removing certain species, but in the sense of preserving soil quality and establishing a buffer zone around the selected area. In the light of our results, the use of *D. viscosa* should be limited, as it could be stabilized as invasive and cause loss of biodiversity and degradation of plant communities. All the aforementioned problems should be considered, especially in areas included within the priority habitat of Coastal lagoons and Mediterranean salt steppes (*Limonietalia*), under the European legislation, where this study has been carried out.

As mentioned above, native-invasive species pose a new challenge for land management in the context of global conservation, so that more detailed studies should be undertaken on this and other native species that may be compromising the conservation of biodiversity.

## Author contributions

MA, JC performed the biochemical assays and the analysis of the data, contributing also to manuscript preparation. OB carried out ion measurements and collaborated in the elaboration of the maps. ED was in charge of the greenhouse work. ML was responsible for sugar quantification by HPLC and, together with MA, performed the DPPH-quenching activity measurements. MD was responsible for the field work and contributed to the elaboration of the maps and figures. OM contributed to the field work and manuscript preparation. OV participated in the general organization and supervision of the work and the interpretation of the results, and was responsible for the final version of the manuscript. MB conceived and designed the study, and participated in writing the manuscript. All authors have read and approved the final version of the manuscript.

## Funding

Work in the UPV laboratories was partly funded by a grant to OV from the Spanish Ministry of Science and Innovation (Project CGL2008-00438/BOS), with contribution from the European Regional Development Fund.

### Conflict of interest statement

The authors declare that the research was conducted in the absence of any commercial or financial relationships that could be construed as a potential conflict of interest.
