# Supplementary material for: Native-Invasive Plants vs. Halophytes in Mediterranean Salt Marshes: Stress Tolerance Mechanisms in Two Related Species
Source: Front Plant Sci. 2016 Apr 18;7:473. doi: 10.3389/fpls.2016.00473 (PMC4834351; doi:10.3389/fpls.2016.00473)
Supplement: Supplementary file 1 [file Table1.DOCX]

**Tab. S1** Electric conductivity (EC_1:5_, dS m^-1^) of substrate samples after 3 and 6-week treatments with the indicated NaCl concentrations, for *D. viscosa* and *I. crithmoides*. Values shown are means ± SD (*n = 5*). For each ion, different letters (lowercase for *D. viscosa* and capital letters for *I. crithmoides*) in a column indicate significant differences between treatments according to Tukey test (α = 0.05). Asterisks (*) indicate significant differences between the two species for the same treatment.

| EC_1:5_ (dS m^-1^) | Period of treatment (weeks) | Treatments  (mM NaCl) | Species | |
| --- | --- | --- | --- | --- |
|  |  |  | *D. viscosa* | *I. crithmoides* |
|  | 3 | 0 | 0,47±0,11a | 0,54±0,16A |
|  |  | 150 | 1,55±0,27b | 1,50±0,34B |
|  |  | 300 | 4,13±0,34c | 4,82±0,75C |
|  |  | 450 | 5,78±0,63d | 5,80±1,02C |
|  |  | 600 | 8,34±1,20e | 7,93±0,96D |
|  | 6 | 0 | 0,44±0,11a | 0,51±0,16A |
|  |  | 150 | 2,92±0,47b | 3,50±0,81B |
|  |  | 300 | 6,02±0,59c | 6,11±1,03C |
|  |  | 450 | 9,30±0,67d | 9,11±0,98D |
|  |  | 600 | 12,5±1,04e | 11,8±1,31E |
